# Verification and comparison of pig, mouse, and human genome similarities: use of manual assembly and analyses

**DOI:** 10.1186/s12864-025-12388-x

**Published:** 2025-12-18

**Authors:** Harry D. Dawson, Celine T. Chen, Jack S. Ragonese, Allen D. Smith, Joan K. Lunney

**Affiliations:** 1 USDA, ARS, Beltsville Human Nutrition Research Center, Diet, Genomics & Immunology Laboratory, Beltsville, MD 20705-2350 USA; 2 USDA, ARS, NEA, APDL, Beltsville Agricultural Research Center, Animal Parasitic Diseases Laboratory, Building 1040, Room 103, BARC-East, Beltsville, MD 20705-2350 USA

**Keywords:** Pig, Mouse, Human genome, Nutrition, Metabolism, Immunity

## Abstract

**Background:**

Recently there have been numerous attempts to improve the genome of the pig. Despite these efforts, there is a substantial amount of work remaining to obtain a “finished version” of the genome; analysis of incomplete versions can lead to incorrect biological interpretations. To that end, we manually assembled and annotated a non-redundant, 16,146 RNA and 15,613 pig protein sequence libraries. We used it to assess the assembly and annotation status of the 3 latest builds of the genome and to the mouse and human genomes.

**Results:**

Our analysis of 6,135 protein-coding genes reveals that the percentage of error-free assembled and annotated genes in NCBI and Ensembl builds 11.1 and MARC build 1.0 are 58.9, 51.7, and 47.1%, respectively. An examination of these errors revealed nine predominant sources that are detailed in the Results. Using our protein library, we determined 1:1 orthology to 16,496 mouse and 15,770 human proteins. 73.8% of these proteins were conserved among the 3 species; however, when a gene was missing from one of the three genomes, pigs were 5.0X more likely to have the human gene than mice. REACTOME, GO BP Direct, and Ingenuity Pathway Analysis functional enrichment analyses of pig-human orthologous genes revealed 8, 13, and 35 conserved pathways, and 0, 0, and 47 for human-mouse pathways, respectively. Last, we conducted an analysis of functional domain preservation for 3,465 proteins and discovered when a functional domain is missing from a protein in 1 of the 3 species, pigs are 2X more likely to have the human domain than mice.

**Conclusions:**

These data strongly indicate that, overall, swine are a scientifically important intermediate species (rodent-human) for conducting scientific research on human health.

**Supplementary Information:**

The online version contains supplementary material available at 10.1186/s12864-025-12388-x.

## Introduction

The human and mouse genomes have undergone automatic and extensive manual annotation by the Human And Vertebrate Analysis and Annotation (HAVANA) group [1]. Recently, the development of “telomere to telomere” sequencing has led to the complete sequencing of the human and mouse genomes [2, 3]. However, there is evidence that the human and mouse genome assemblies are still flawed and could benefit from reassembly; at least 87 human and 22 mouse genes have their NCBI Annotation category listed as “suggests misassembly”. In contrast, only 21 pig genes have their NCBI Annotation category listed as “suggests misassembly”. There have been numerous attempts to improve the annotation and assembly of the porcine genome [4–8]. Despite these efforts, several recent analyses indicates that there is a significant amount of work that needs to be done towards a “finished” pig genome [9, 10], particularly the need for manual annotation [6, 8].

Although recent work by our group and others overwhelmingly suggests that pigs and humans exhibit greater genome similarity at the macro level and share more genes [11–13], our preliminary analysis indicated that pigs and humans have greater conservation of protein functional domains [11], than do humans and mice. A recent study concluded that humans and mice share more Kyoto Encyclopedia of Genes and Genomes (KEGG)-related pathways than humans and pigs [14]; however, the authors of that study speculated that their conclusion is likely to be affected by the incomplete status of the annotations in the porcine genome.

The various pig genomes annotated by NCBI and Ensembl (Table 1) have a wide range of predicted genes (46573–152168), predicted protein-coding genes (19974-22125), predicted transcripts (56900–78200) and predicted transcripts/gene ratio (0.39–2.92). It is not logical to assume that there is this much natural, pig breed variation. But rather, this variation is likely pipeline intrinsic and due to the variable amounts of updates and patches applied to each. This assertion is supported by the observation that, using the same sequence source (Duroc build 11.1), NCBI predicts 6.8x and 2.9x more pseudogenes and transcripts per gene, respectively, than Ensembl. Furthermore, a recent paper using automated analysis has noted the incongruity of the annotation of protein-coding porcine genes in the NCBI and Ensembl builds of 11.1 with 2119 and 3371 discordant genes [15]. Potential sources of this discrepancy were not identified. These data make it difficult to assess the actual number of genes and transcripts in machine-annotated genomes. Furthermore, any cross-species functional comparison using pigs would be compromised by these discrepancies.

**Table 1:**
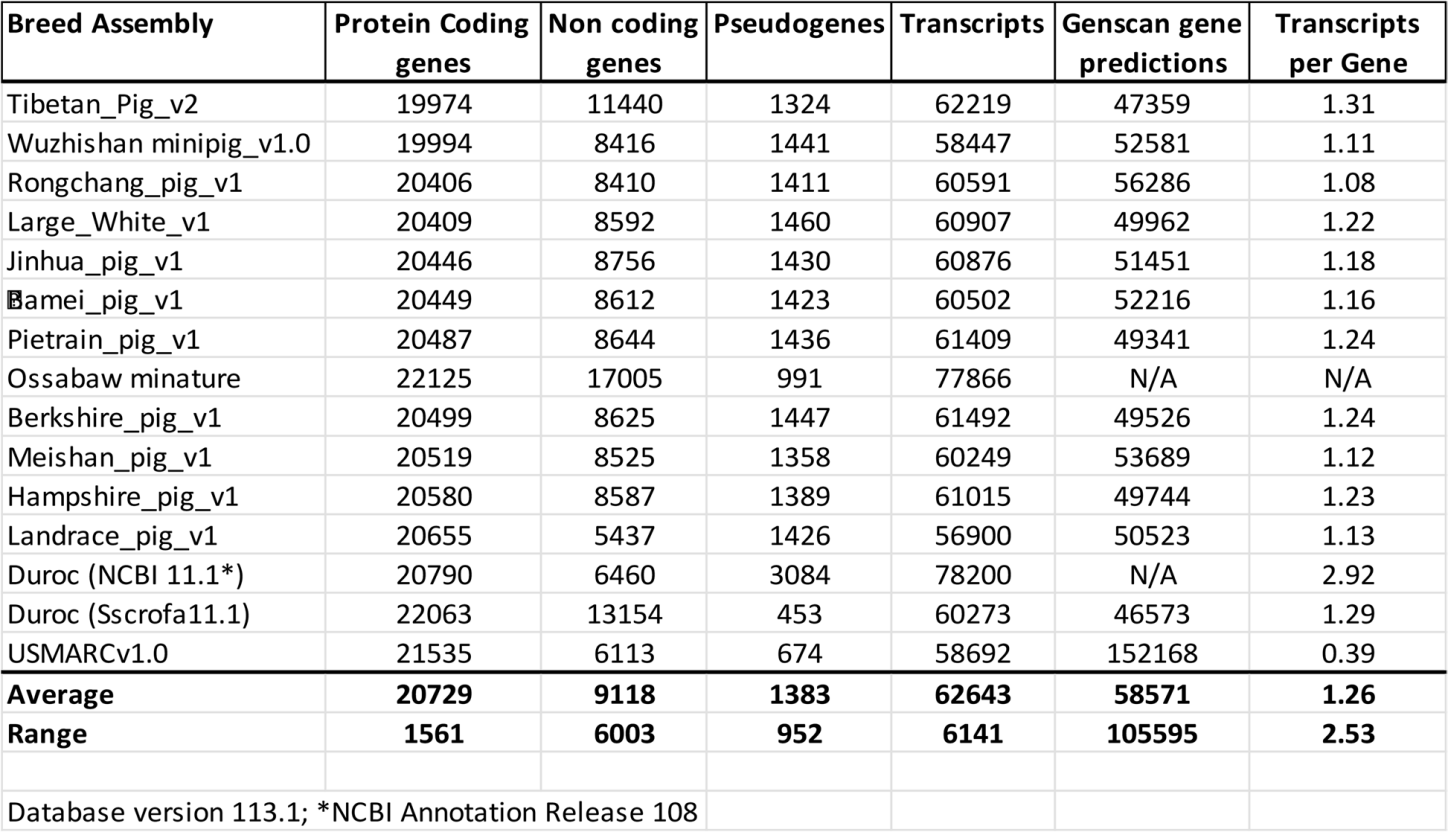
Comparison of pig genome assemblies

Previously we discovered several sources of systematic errors in the earlier pig reference genome (Ensembl and NCBI builds 10.2) prediction or annotation pipelines (selenoproteins, taste receptor genes, intronless genes, artifactually duplicated genes) by using a manually annotated set of sequences [16]. Herein, we extend our analysis to Ensembl and NCBI builds 11.1 and MARC 1.0, with a much larger manually assembled and annotated library of RNA and protein sequences (16,146 non-redundant RNA and 15,613 pig protein sequences), in order to uncover potential systematic errors. We used this library to determine the conservation of pig and human 5’ and 3’UTR (untranslated regions) RNA regions and RNA splice variants. We then used this nonredundant and highly annotated pig proteome to identify 1–1 mouse and/or human orthologs. We compared the 1–1 orthologs for all 3 species to determine functional enrichment. A similar analysis was performed on proteins with shared or non-shared functional domains.

## Results

### Error Analysis and Categorization

####  Protein-coding Genes

In a comparison of a subset of the 18,405 protein-coding genes (6,135 from each genome), we determined that the percentage of correctly assembled and annotated genes in the NCBI build 11.1, Ensembl build 11.1 and MARC build 1.0 to be 58.9%, 51.7% and 47.1%, respectively (Table 2). The sources of errors were varied. The most frequent broadly defined error category, error in annotated locus that includes assembly errors, gene prediction errors, annotation tool discrepancies, reference genome bias, poor data quality, repetitive sequences, manual curation errors and chimeric gene mis-annotations, occurred in 24.9, 42.6, and 43.8%, of NCBI build 11.1, Ensembl build 11.1 and MARC build 1, respectively. Because of the higher error rate of MARC 1.0, we examined a larger, randomly chosen set (9915 genes) in the Ensembl assembly of MARC 1.0, compared in Table 2. The expanded search of MARC 1.0 revealed a similar error rate (53.3% vs. 52.9%) and identified additional missing genes (306 of which 265 are protein-coding) compared to NCBI (28) and Ensembl build 11.1 (36) (Tables 2 and 1S). The missing genes span approximately 99.2 Mb of the genome and involved significant segments of porcine chromosomes 1 (41 genes), 2 (41 genes) and 13 (27 genes). This missing gene rate is much higher than expected. These areas of MARC 1.0 should be targeted for resequencing in any future builds. Our analysis discovered 582 protein-coding genes that are not annotated in MARC 1.0. If these results were extended to the whole genome, over 1,100 protein-coding genes would not be annotated and 500 would be missing. We also examined 500 genes from the newly sequenced Ossabaw genome (build 1.0 deposited in Ensembl) and found the error rate (50.2% versus 52.9%) that was very similar to that of MARC build 1.0 (Table 2).

**Table 2:**
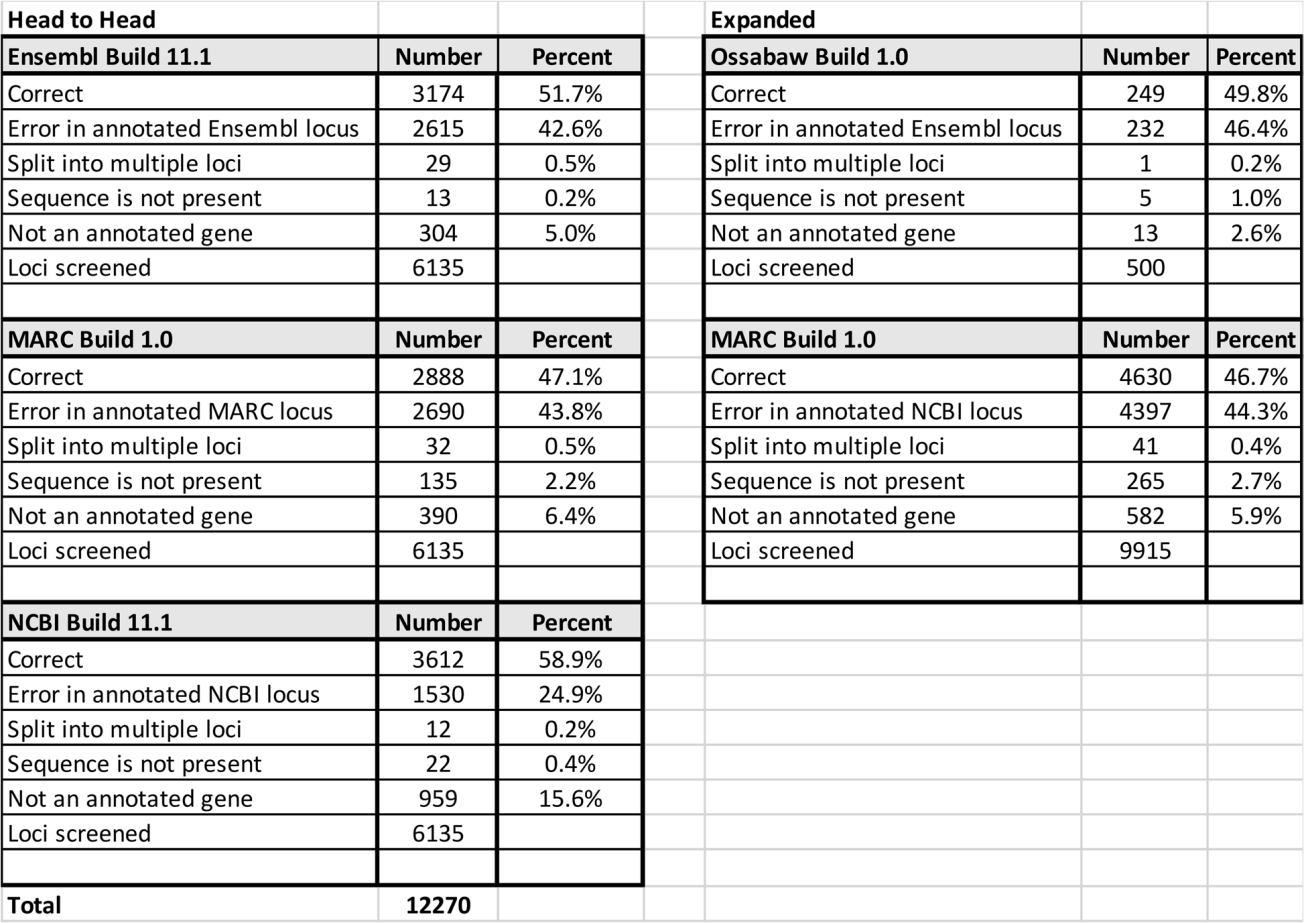
Summary of 3 genomes’ build errors (protein-coding genes)

#### Proteins of extreme size/indel analysis

We analyzed 350 proteins of extreme size (> 2,000 amino acids (AA)) (Table 2S). Our error analysis revealed that the percentage of correctly assembled genes for these proteins in NCBI build 11.1, Ensembl build 11.1 and Ensembl MARC build 1.0 is, respectively, 53.1%, 27.4% and 23.4%. This analysis also identified the most serious source of error that prohibited correct assembly and annotation of Ensembl and NCBI builds of 11.1, was the presence of an indel every 12,465 bp. This is rather surprising since the coverage of the genome averaged 65x [8]. Using the search term “low quality protein” in NCBI, yields 2807 porcine protein-coding genes that are affected by this. This number of proteins is inflated because some of these low-quality proteins are pseudogenes; however, the number is likely to be much higher in Ensembl build 11.1 because of the presence of pre-genome, annotated reference sequences in NCBI build 11.1. NCBI usually fills the indel with ambiguous nucleotides (N), as a place holder and annotates it as a correctly sized, but low-quality protein. Ensembl does not do this. As a result, a great number of truncated or elongated proteins arise in the Ensembl assembly because the algorithm appears to be searching for the next best splicing site. These genes may still be useful when doing RNA-Seq if the stringency of matching is lowered; however, this raises the risk of erroneously mapping high-similarity sequences. As expected, every protein-coding gene we evaluated and identified in NCBI build 11.1 as low quality (1437) was also incorrect in Ensembl build 11.1; however, only 28.7.1% of these proteins (412) were correctly assembled in MARC, reflecting the overall fundamental error in the gene assembling algorithm, particularly with genes that have a high number of exons.

This error also limits the accuracy of splice variant determination, as NCBI does not annotate predicted splice variants of low-quality proteins, and functional domain analysis, so many of the misassembled proteins are missing one or more functional domains. Last, although the insertion of an indel by NCBI benefits the analysis of genes affected by a real indel, NCBI also inserts an ambiguous nucleotide(s) and annotates pseudogenes as low-quality proteins in the presence of a predicted stop codon. We found that 218/1582 (13.8%) of NCBI-annotated pig low-quality genes were actually pseudogenes.

#### Intronless Genes

Intronless genes constitute a significant number of genes with errors. Intronless genes make up approximately 3% of the human genome [17]. These genes can be divided into 2 categories, genes that consist of a single exon (true intronless genes) and genes whose protein-coding region span a single exon but are interrupted by introns in the UTRs. Estimates of the number of human single exon coding region genes approaches 2000 [18]. The number of intronless genes in the pig is likely to be much higher because of the large number of Olfactory Receptor (OR) genes.

Previous analysis of the intronless genes in humans and mice revealed that automatic annotation of these genes is problematic and manual annotation or specialized, supervised pipelines have been used to determine various classes of intronless genes [19–22]. Two related protein superfamilies, keratin associated proteins (KRTAP) and late cornified envelope (LCE) proteins are overrepresented in intronless genes. Other prominent classes of protein-coding genes overrepresented in intronless genes are G-protein coupled receptors. Subclasses of genes found in intronless G-protein-coupled receptors include vomeronasal receptors (VMRs) and TRs.

##### Keratin associated proteins (KRTAP) and late cornified envelope (LCE) proteins

Our study determined that humans have 124 (107 genes, 17 pseudogenes), pigs have 125 (110 genes and 15 pseudogenes) and mice have 187 (141 genes and 46 pseudogenes) *KRTAP* genes (Tables 3 and 3S). The vast majority of the porcine genes we identified are not annotated genes in NCBI 11.1 (80 missing), Ensembl 11.1 (64 missing) or MARC 1.0 (68 missing) genomes (Table 3S). Furthermore, they have very limited sequence homology, so assigning 1:1 orthology is difficult. Our study also determined that humans have 19, pigs have 15 and mice have 21 LCE proteins. Like the KRTAP genes, many of the 15 porcine LCE genes we identified are not annotated genes in NCBI 11.1 (10 missing), Ensembl 11.1 (7 missing) or MARC 1.0 (8 missing) genomes.

**Table 3:**
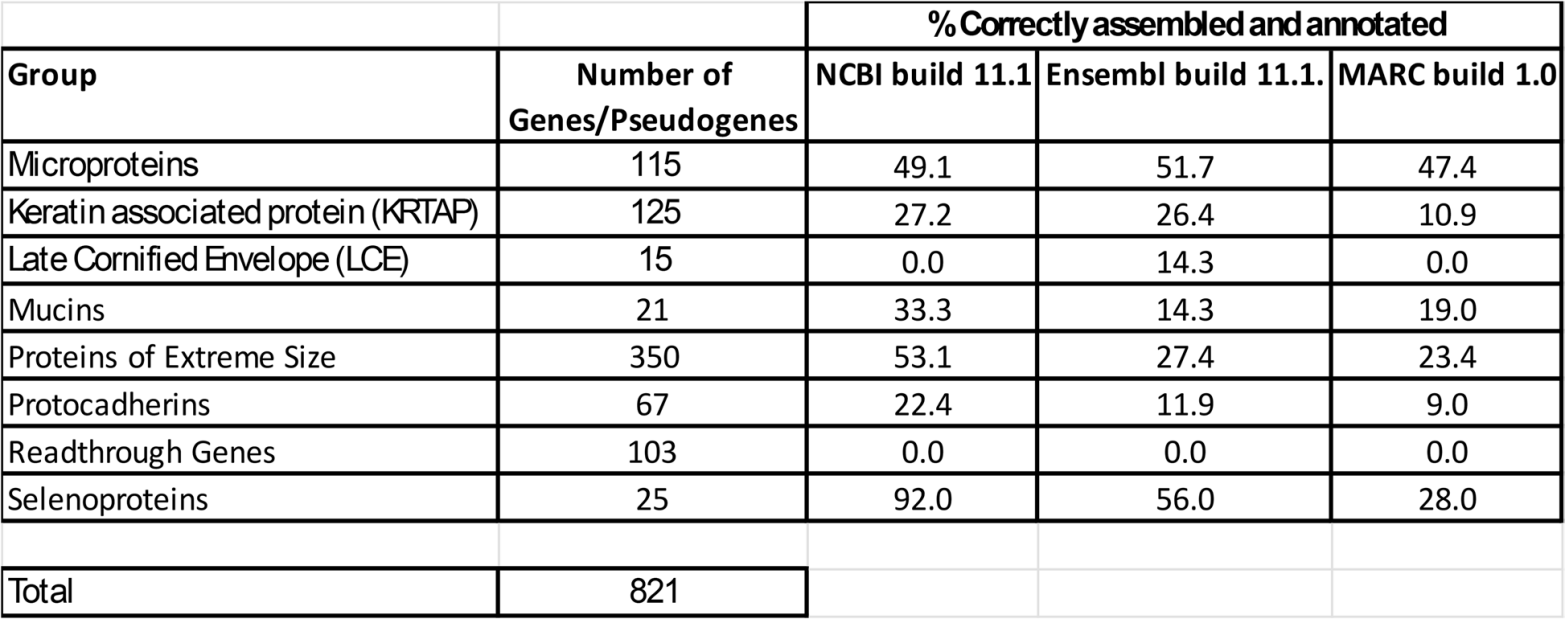
Comparison of 3 Swine Genome Assemblies

##### Vomeronasal receptors (VMRs)

We found that pigs (14) and humans (5) have a significantly smaller number of VMRs (VMN1 + VMN2) compared to mice (225). The larger number of VMR genes in pigs relative to humans is because the pig VN1R4 gene has diverged into 12 paralogs (VN1R4, VN1R4L1, VN1R4L2, LOC110261363, LOC110261366, LOC110261370, LOC110261364, LOC102167894, LOC100520313, LOC100738896, LOC106510602) and 2 pseudogenes (VN1R4Ps1, VN1R4Ps2). We identified one intact pig-mouse VMN1 ortholog (VMN1R233). VMN2R1, a rodent-specific VMN2 gene, is an expressed pseudogene in pigs.

#### Endogenous Retroviral Sequences (ERVs) and Retrotransposons

We discovered more than 750 endogenous retroviral and retrotransposon-associated sequences (reverse transcriptase, transposases), spanning over 3 million bps in Ensembl build 11.1 (Table 4S). Some of them are annotated as protein-coding, very few of them are correctly annotated. Previous studies have reported that LINE 1 (L1) and Line 2 elements account for 18.5% of the pig genome [23]. LINE 1 elements are highly abundant poly(A) (non-LTR) retrotransposons whose second open reading frame (ORF2) encodes a reverse transcriptase (RT) and a transposase (TR) [24]. In pigs, the intact sequence for the representative RNA/protein RT sequence EF599954.1/ ABR01162.1 (1272 AA), is found over 130 times in the NCBI/Ensembl build 11.1 genomes, there also are hundreds of partial sequences. These are widely distributed across the genome, occurring on every chromosome. Previously the chromosome X located RT gene was described as interrupting the KPL2 gene in Finnish Yorkshire pigs [25]. We have found that the RT gene frequently occurs in the intron or non-coding exon of many different protein coding genes. The conceptual RT ORFs found in the pig genome are infrequently annotated as protein-coding, but only 18 full length copies are found in Ensembl build 11.1 as annotated genes. Many others contain stop codons and indels or are improperly annotated as multiexonic, that lead to shorter versions or LncRNA annotation. When Ensembl annotates these genes, the number of predicted exons range from 1 to 7. NCBI does not annotate them, except accidently as an exon of the 6-phosphofructo-2-kinase/fructose-2,6-bisphosphatase 1 gene (XP_020935075.1). These RT genes are found exclusively in build 11.1 of the Duroc genome and not present in other Ensembl-curated pig genomes (Bamei, Berkshire, Hampshire, Jinhua, Meishan, Landrace, Large White, Pietrain, Rongchang, Tibetan, Wuzhishan or Ossabaw). It is unclear to us why they are unique to the Duroc genome and why they have been filtered out of other Ensembl-curated genomes.

An estimated 80–100 active L1 retrotransposon elements (out of several thousand present) [24] are found throughout the average human genome, but only one protein (L1TD1, 865 AA), located on chromosome 1, is annotated as a transposase as part of the protein coding genome by NCBI. The NCBI-annotated L1TD1 pig protein (XP_020935846.1, Uniprot A0A5G2QE83) is 264 AA protein, located on chromosome X, is probably not the 1:1 ortholog. Furthermore, this sequence is missing from the MARC 1.0 and Ossabaw 1.0 genome builds. Several hundred conceptual ORF DNA sequences for a series of LINE-1 (L1) type transposase domain-containing proteins (ABR01161.1, 296 AA, Uniprot A5Z0F5), occur in Ensembl build 11.1 of the pig genome; however, only 56 loci are annotated as intact 296 AA protein-coding genes. We have annotated the group of TR proteins as followed by the chromosome number. This protein was also previously described on chromosome X and interrupting the KPL2 gene in Finnish Yorkshire pigs [25]. It occurs in numerous genes in Ensembl build 11.1, but only 1 is erroneously annotated as a protein coding exon of the IMMT gene in Ensembl build 11.1 and Ossabaw build 1.0. Other than that, Ossabaw build 1.0 and NCBI build 11.1, do not annotate them as an intact 296 AA protein-coding genes. There are several hundred conceptual ORF in MARC build 1.0, but only 14 are annotated as intact protein coding, TR genes. There are variable number of intact, non-overlapping, 296 AA (Uniprot A5Z0F5) protein-coding, TR genes occurring in other Ensembl-curated pig genomes at various numbers; Bamei (8), Berkshire (6), Hampshire (8), Jinhua (8), Meishan (10), Landrace (2), Large White (4), Pietrain (4), Rongchang (6), Tibetan (18), Wuzhishan (46).

There are another series of higher molecular weight, 301 AA TR proteins (HMW TR, Uniprot A0A8W4FHG9), that are not found as an intact protein coding gene in the NCBI NR protein database (it is present in the Transcriptome Shotgun Assembly (TSA) protein database). They are present in Ensembl build 11.1 (2) and in other Ensembl-curated pig genomes at variable sizes and numbers. A related, fourth series of lower molecular weight, 199 AA TR proteins (LMW TR) (Uniprot A0A480WDB1) are also not found as an intact protein coding gene in the NCBI NR protein database (they are present in the TSA protein database. Like the HMW TR proteins, they are present in Ensembl build 11.1 (2) and in other Ensembl-curated pig genomes at variable sizes and numbers. Although they appear in the TSA archives (presuming they are transcribed) it is unknown how many active L1 retrotransposon elements are found throughout the pig genome, so it is difficult to determine what genes to annotate as truly protein-coding. Nevertheless, the wide discrepancy in annotated status of these genes in the various pig genomes as well as other mammalian genomes significantly contributes to the publicized differences in the number of protein coding genes found in the various sequenced pig genomes as well as differences between pigs and other species.

Another contributor to the difference between the number of protein coding genes in sequenced pig genomes is the annotation of various endogenous retrovirus (ERVs) sequences as protein coding. There are multiple partial or full copies of the PERV genome in build 11.1 of the genome but it is unknown whether some or all are transcribed. Previously 26 locations have been described for PERV, including 5 non-reference PERV sequences, scattered throughout the 11.1 reference genome [26, 27]. We discovered multiple intact or partial protein sequences for group-specific antigen (gag, 524 AA), polymerase (pol, 1144 AA), and envelope (env) (PERV A/C (657 AA). In addition to genes belonging to Porcine Endogenous Retrovirus (PERV)-A/C, we identified genes from other retroviruses such as endogenous retrovirus group V member 1 (ERVV-1), Env polyprotein-like, ERV3-1-like genes, ERVK − 19-like gene (a 135 AA protein that is weakly homologous to pig reverse transcriptase-like protein AAX77009.1) and ARTgagpol, a 1267 AA ancient retroviral gagpol previously described in artiodactyla [28]. ERVs comprise 8% of the human genome [29]; however, few are translated into functional proteins. It is unknown whether any/all of these pig ERV sequences can be expressed and whether they should be considered part of the protein coding genome. These potential artifacts significantly inflate the number of pig proteins in the Ensembl build 11.1, especially those that have been deemed to be pig specific. The vast majority of these are filtered out of the human and mouse Ensembl genome builds.

#### Selenoproteins

In NCBI build 11.1, only one selenoprotein protein is incorrectly assembled, however; in Ensembl build 11.1 and MARC build 1.0 of the porcine genome, 11 out of 25 (44%) and 18 out of 25 (72%), respectively, of selenoprotein genes are assigned a premature stop codon or have additional errors (Tables 3 and 4S). All human and mouse proteins are correctly assembled and annotated in NCBI and Ensembl.

#### Mucins

We identified 20 mucin genes in pigs. Of these, 33.3%, 14.3% and 19.0% are properly assembled and annotated in NCBI build 11.1, Ensembl build 11.1 and MARC build 1.0, respectively, and only two genes, CD164 and MUC15, were assembled properly in all three builds. There is, however, little overlap in this gene set (Tables 3 and 6S) with a variety of assembly errors throughout all three builds.

#### Protocadherins

Between the 3 species, we identified 80 protocadherin genes. Sixty-seven protein-coding protocadherin genes exist in pigs. Of these, 22.4%, 11.9% and 9.0% are properly assembled and annotated in NCBI build 11.1, Ensembl build 11.1 and MARC build 1.0, respectively; however, there is little overlap in this gene set. (Tables 3 and 7S).

#### Readthrough, fusion or conjoined genes

Via manual annotation, we determined that pigs can likely make 103 readthrough genes (Tables 3 and 8 S). We developed a scoring system where we assessed the confidence of our predictions. The categories from lowest to highest confidence are; 0 = no transcript could be predicted, human, primate or mouse-specific gene; 1 = Predicted transcript but no evidence of existence in other species; 2 = Predicted transcript, limited evidence of existence (2 species or less); 3 = Predicted transcript, limited evidence of existence (2 species or less), transcription demonstrated in pig; and 4 = Predicted transcript, evidence of existence (2 species or more), transcription demonstrated in pig. The number of pig readthrough genes in each category are 7, 30, 42 and 24 for categories 1, 2, 3 and 4, respectively. Ninety two of these correspond to human or primate genes and many have orthology to transcripts in other mammalian species. We determined that there are only three pig genes annotated as readthrough genes in NCBI build 11.1, Ensembl build 11.1 and MARC build 1.0, combined.

#### Microproteins

Microproteins and small open reading frames (sORFs)-encoded proteins (SEPs) are small (< 100 AA) proteins containing a single domain. Standard genome annotation pipelines routinely miss these [30]. They can exist as distinct genes or arise as a result of a shift in the open reading frame during translation. More than 1,000 of these have been identified in mice and humans, using the OpenProt 2.0 database [31]. Sheep and cow proteins, but not pig proteins, are indexed in this database. We identified 153 human microproteins via a literature search as these genes are sometime not annotated as protein-coding genes in the OpenProt or NCBI-annotated genome. We discovered 115 pig orthologs or paralogs of these genes by translated BLAST (tblastn) of the human protein sequence to the pig genome. The corresponding DNA sequence was translated, and the putative DNA or protein sequences were used to search whether these proteins were annotated as such in the 3 porcine genomes. Data is presented in Tables 3 and 9S. Only 49.1%, 51.7% and 47.4% pig microprotein RNA or proteins are properly assembled and/or annotated in NCBI build 11.1, Ensembl build 11.1 and MARC build 1.0, respectively,

### Protein Orthology and Functional Domain Analysis

#### Protein Orthology Analysis

**Fig. 1:**
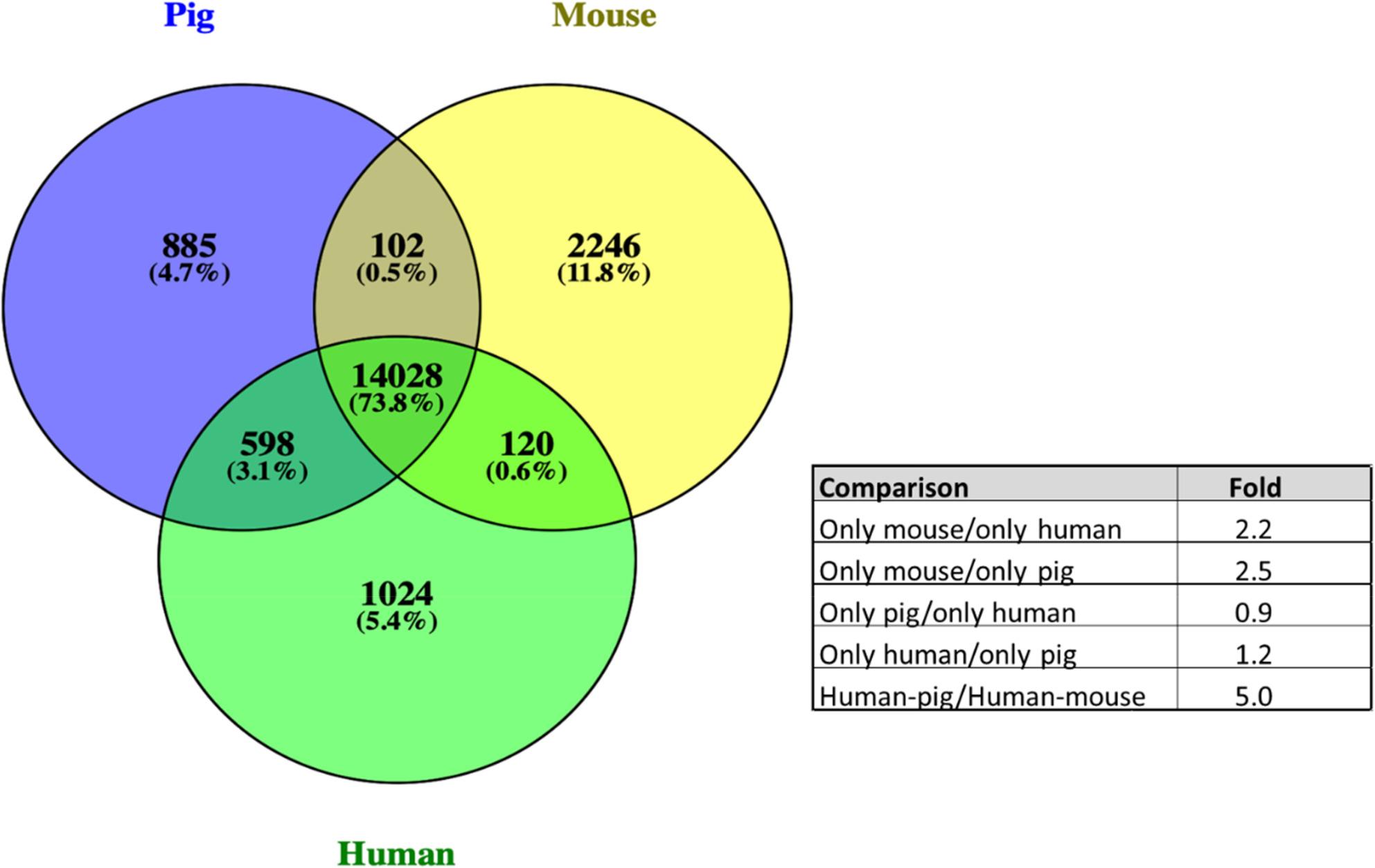
Greater Pig-Human Similarity of Genes Revealed by Analysis of Non-conserved Protein-coding Genes. Venn diagrams were prepared using Venny 2.1. (http://bioinfogp.cnb.csic.es/tools/venny/index.html). When a gene is missing from one of the 3 genomes, pigs are 5.2 X more likely to have the human gene than mice

We determined orthology for 47,879 (15,613 pig, 16,496 mouse, and 15,770 human) protein-coding genes (Table 10 S and Fig. 1). NCBI lists 20,790, 22,192 and 20,080 protein-coding genes in pigs, mice, and humans, respectively; therefore, our analysis is estimated to encompass 75.1% of pig, 74.3% of mouse, and 78.5% of human proteins (a species average of 76.0%). We omitted OR, TCR and BCR, and MHC class I and II proteins from our analysis. Our species average estimate of coverage of the proteome excluding these groups is likely to exceed 80%. Our analysis shows that when a gene is missing from one of the three genomes, pigs are 5.0 × (598/120) more likely to have the human gene than mice (Table 10 S, Summary Sheet). Mice had 2.2 (2246/1024) and 2.5 × (2246/885) the number of unique proteins compared to humans and pigs, respectively (Table 10 S, Summary Sheet). Some of these unique proteins, and their pseudogenes, were categorized by Superfamily and appear in Table 4. As expected, mice exhibited the largest number of Superfamily member expansions.

**Table 4:**
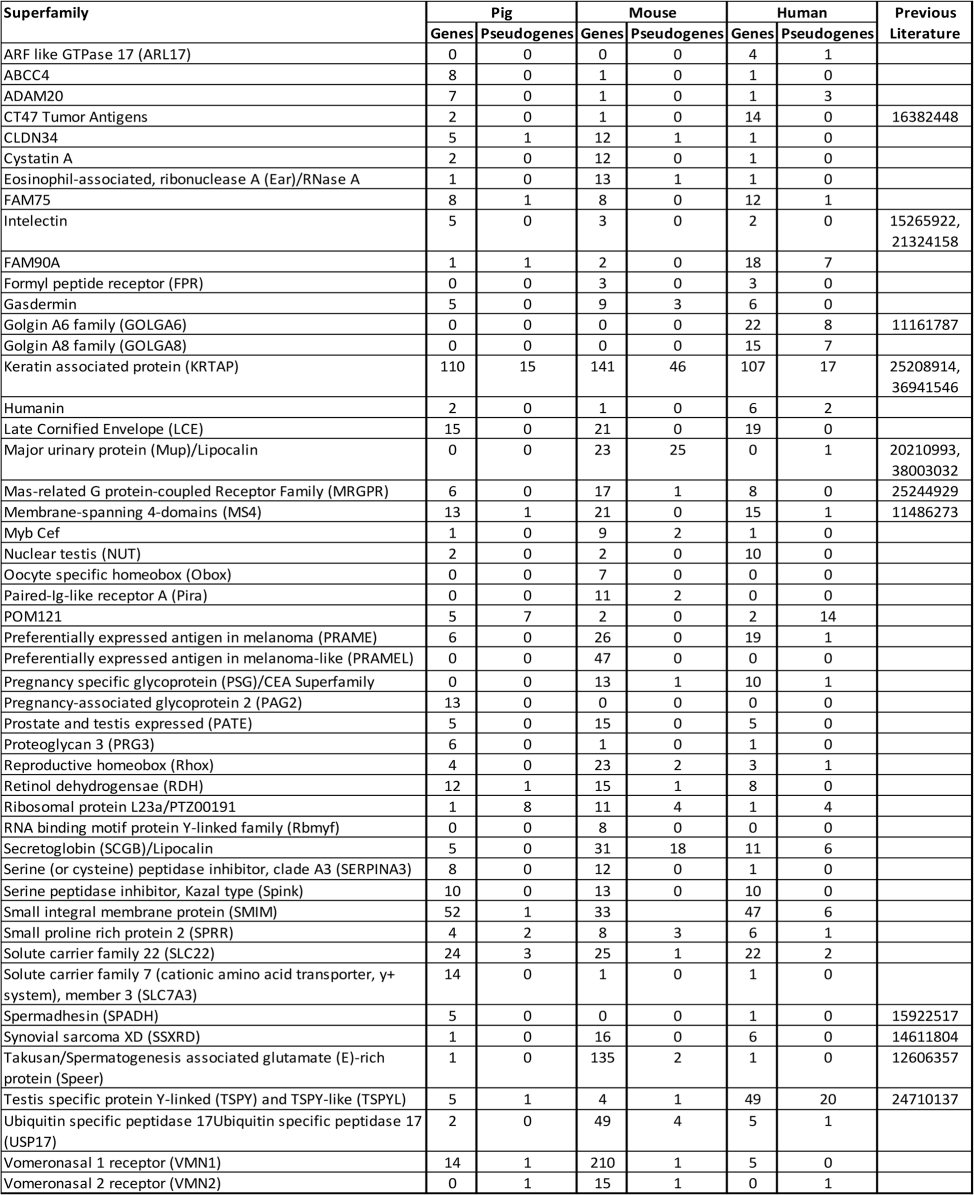
Expanded Superfamilies

We conducted DAVID data mining of differentially encoded proteins to determine pathway enrichment. We found 8 REACTOME pathways (Tables 5 and 10S) that were enriched after correction for multiple comparison, for the pig-human comparison; R-HSA-212,436 ~ Generic Transcription Pathway (3.0 fold, *p* = 5.27E-24), R-HSA-6,805,567 ~ Keratinization (7.1 fold, *p* = 5.67E-22), R-HSA-73,857 ~ RNA Polymerase II Transcription (2.7 fold, *p* = 3.61E-21), R-HSA-74,160 ~ Gene expression (Transcription) (2.4 fold, *p* = 8.95E-18), R-HSA-1,461,957 ~ Beta defensins (13.2 fold, *p* = 4.91E-12), R-HSA-6,803,157 ~ Antimicrobial peptides (7.7 fold, *p* = 5.09E-11), R-HSA-1,461,973 ~ Defensins (10.8 fold, *p* = 1.24E-10) and R-HSA-168,249 ~ Innate Immune System (1.5 fold, *p* = 3.93E-02). We found no significant pathway enrichment for the mouse-human REACTOME comparisons.

**Table 5:**
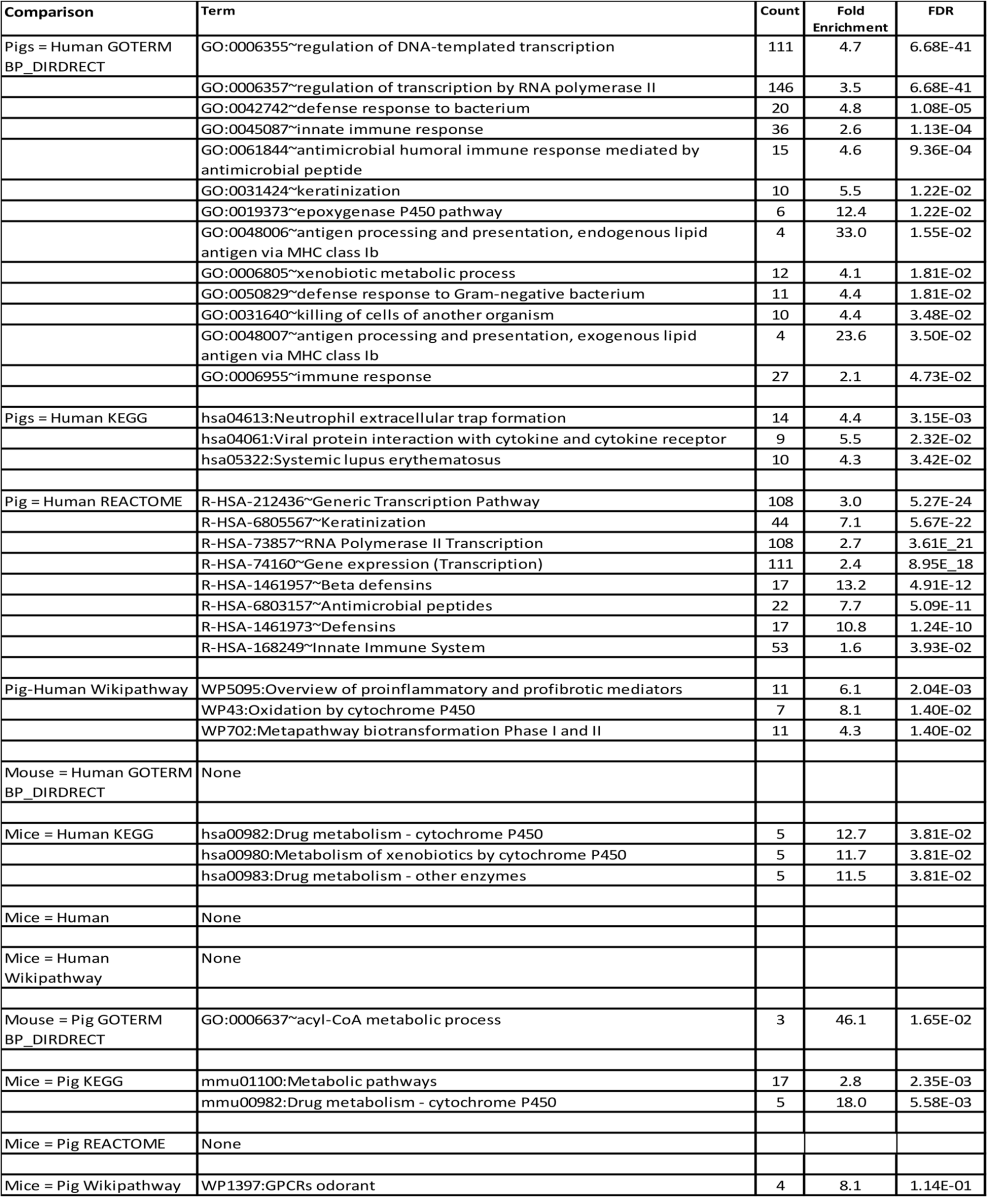
Analysis of Differentially Encoded Proteins to Determine Pathway Enrichment

We conducted GO BP DIRECT mining of differentially encoded proteins to determine ontology enrichment. We found 13 GO terms that were enriched after correction for multiple comparison for the pig-human comparison, although many more pathways had p values < 0.05 before correction (Table 5). GO:0006355 ~ regulation of DNA-templated transcription (4.7 fold, *p* = 6.68E-41), GO:0006357 ~ regulation of transcription by RNA polymerase II (4.7 fold, *p* = 6.68E-41), GO:0042742 ~ defense response to bacterium (3.5 fold, *p* = 6.68E-41), GO:0045087 ~ innate immune response (2.6 fold, *p* = 1.13E-04), GO:0061844 ~ antimicrobial humoral immune response mediated by antimicrobial peptide 4.6 fold, *p* = 9.36E-04), GO:0031424 ~ keratinization (5.5 fold, *p* = 1.22E-02), GO:0019373 ~ epoxygenase P450 pathway (12.4 fold, *p* = 1.22E-02), GO:0048006 ~ antigen processing and presentation, endogenous lipid antigen via MHC class Ib (33.3 fold, *p* = 1.55E-02), GO:0006805 ~ xenobiotic metabolic process (4.1 fold, *p* = 1.81E-02), GO:0050829 ~ defense response to Gram-negative bacterium (4.4 fold, *p* = 1.81E-02), GO:0031640 ~ killing of cells of another organism (4.4 fold, *p* = 3.48E-02), GO:0048007 ~ antigen processing and presentation, exogenous lipid antigen via MHC class Ib (23.6 fold, *p* = 3.50E-02), GO:0006955 ~ immune response (2.1 fold, *p* = 4.73E-02). We found no significant pathway enrichment for the mouse-human GO BP DIRECT comparisons.

**Fig. 2:**
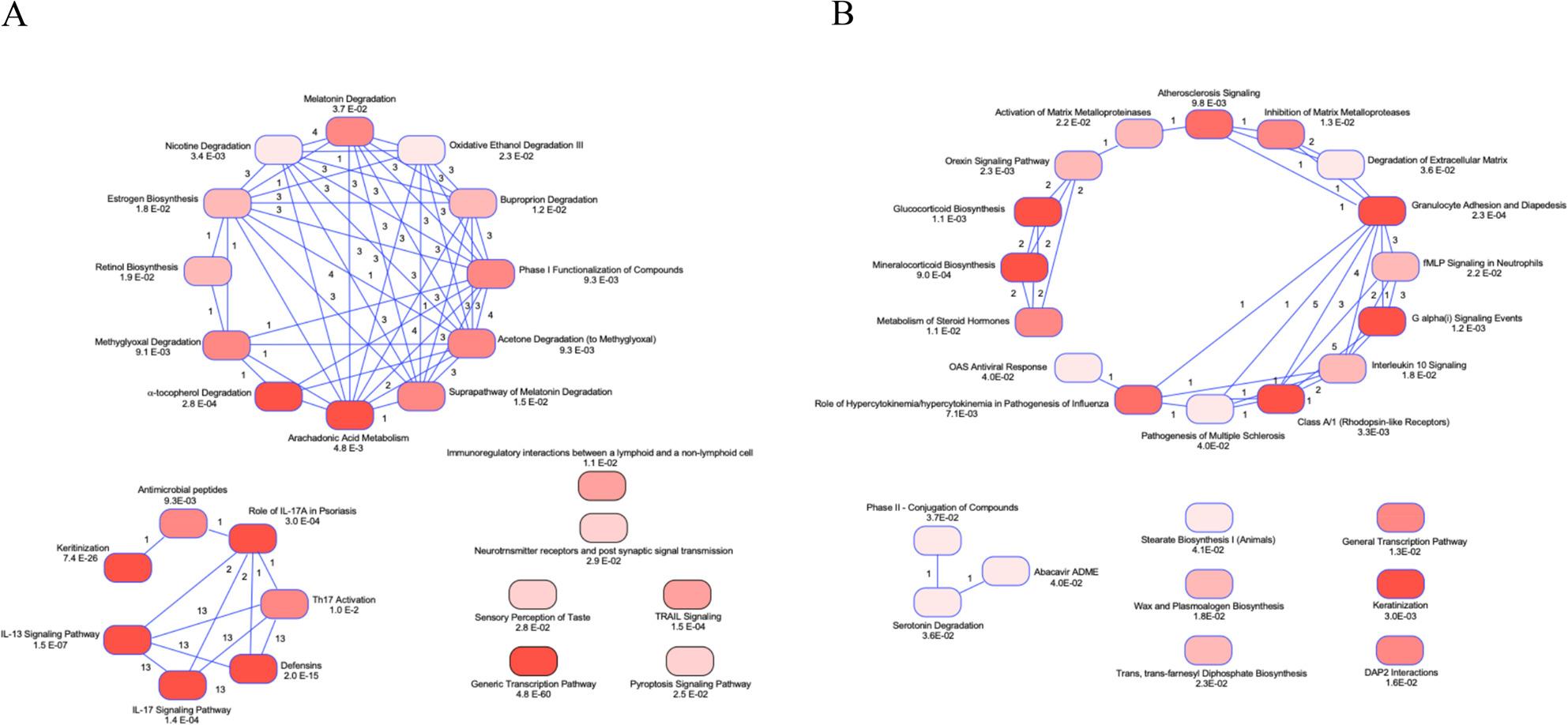
Analysis of Pig-Human (**A**) or Mouse-Human (**B**) Conserved Pathways. Ingenuity Pathway Analysis functional enrichment revealed 32 and 29 enriched pathways for **A**. pig-human or **B**. mouse-human, orthologous genes, respectively. Each line represents a known biological relationship between the two pathways. The numbers next to the relationship lines represent the number of supporting scientific findings from the literature or canonical information in the Ingenuity Pathways Knowledge Base

We conducted Ingenuity Pathway Analysis (IPA) of pig-human and mouse-human conserved genes and found 32 and 29 pathways respectively. These are summarized in graphical form in Fig. 2 and in tabular form in Table 11S. In addition to the pathways discovered by REACTOME and GO BP Direct, genes involved in IL-13 Signaling Pathway (*p* = 1.48E-07) and IL-17 Signaling pathway (*p* = 1.43E-04) as well as Retinol Biosynthesis (*p* = 1.93E-02) and α-tocopherol Degradation (*p* = 2.79E-04) were enriched in the pig-human dataset (Fig. 2A). For the mouse-human dataset (Fig. 2B), Granulocyte Adhesion and Diapedesis, the top canonical pathway (*p* = 9 2.27E-04) and Interleukin-10 signaling (*p* = 1.78E-02) were the sole immune-related pathways. There was no enrichment of nutrition related pathways determined by IPA. Although there were similar numbers of pig-human and mouse-human conserved pathways (45 mouse-human pathways were significant at a *p* < 0.01; whereas 35 pig human pathways were significant at a *p* < 0.01), the number of genes per node and the statistical significance was less for the mouse-human comparisons.

#### Functional Domain Comparison

**Fig. 3:**
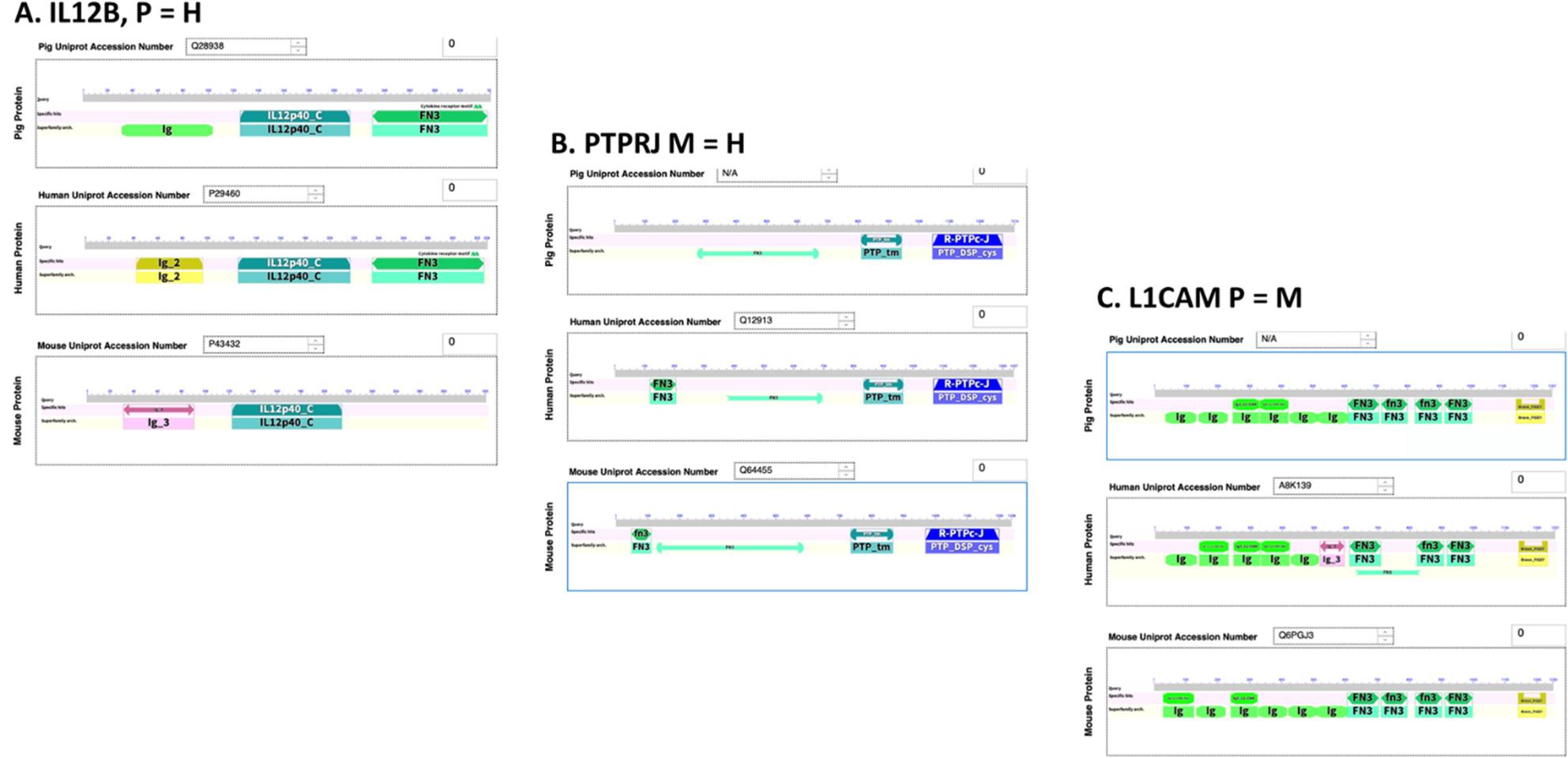
Corresponding Protein Isoforms with Non-conserved FN3 Functional Domains. Two-dimensional analyses were generated using NCBI Blast. Results showed greater Pig Human similarity occurs for **A**) for IL12B. Greater Mouse Human similarity occurs for **B**) PTPRJ proteins. Greater Pig-Mouse similarity occurs for **C**) L1CAM protein

We examined a randomly chosen subset of 3465 protein-coding genes (10395 proteins overall) for preservation of protein Superfamily domains or other features (Tables 4 and 11 S). Three examples of these are shown in Fig. 3. We identified 644 structural differences between 1737 proteins (shared between pig, mouse, and human). We then conducted DAVID data mining of differentially expressed functional domains to determine pathway enrichment (Table 6). We found 2 REACTOME pathways that were enriched after FDR correction for multiple comparison, for the pig-human comparison R-HSA-1,474,244 ~ Extracellular matrix organization (4.8 fold, *p* = 1.22E-04) and R-HSA-168,256 ~ Immune System (1.9 fold, *p* = 4.46E-03). We found 4 pathways that were significantly enriched in the pig-mouse-comparison; R-HSA-168,256 ~ Immune System (2.3 fold, *p* = 1.35E-05), R-HSA-168,898 ~ Toll-like Receptor Cascades (7.7 fold, *p* = 1.15E-04), R-HSA-1,280,215 ~ Cytokine Signaling in Immune system (3.2 fold, *p* = 3.76E-04) and R-HSA-449,147 ~ Signaling by Interleukins (3.5 fold, *p* = 1.17E-02). In contrast, we found no pathway enrichment for the mouse-human comparison.

**Table 6:**
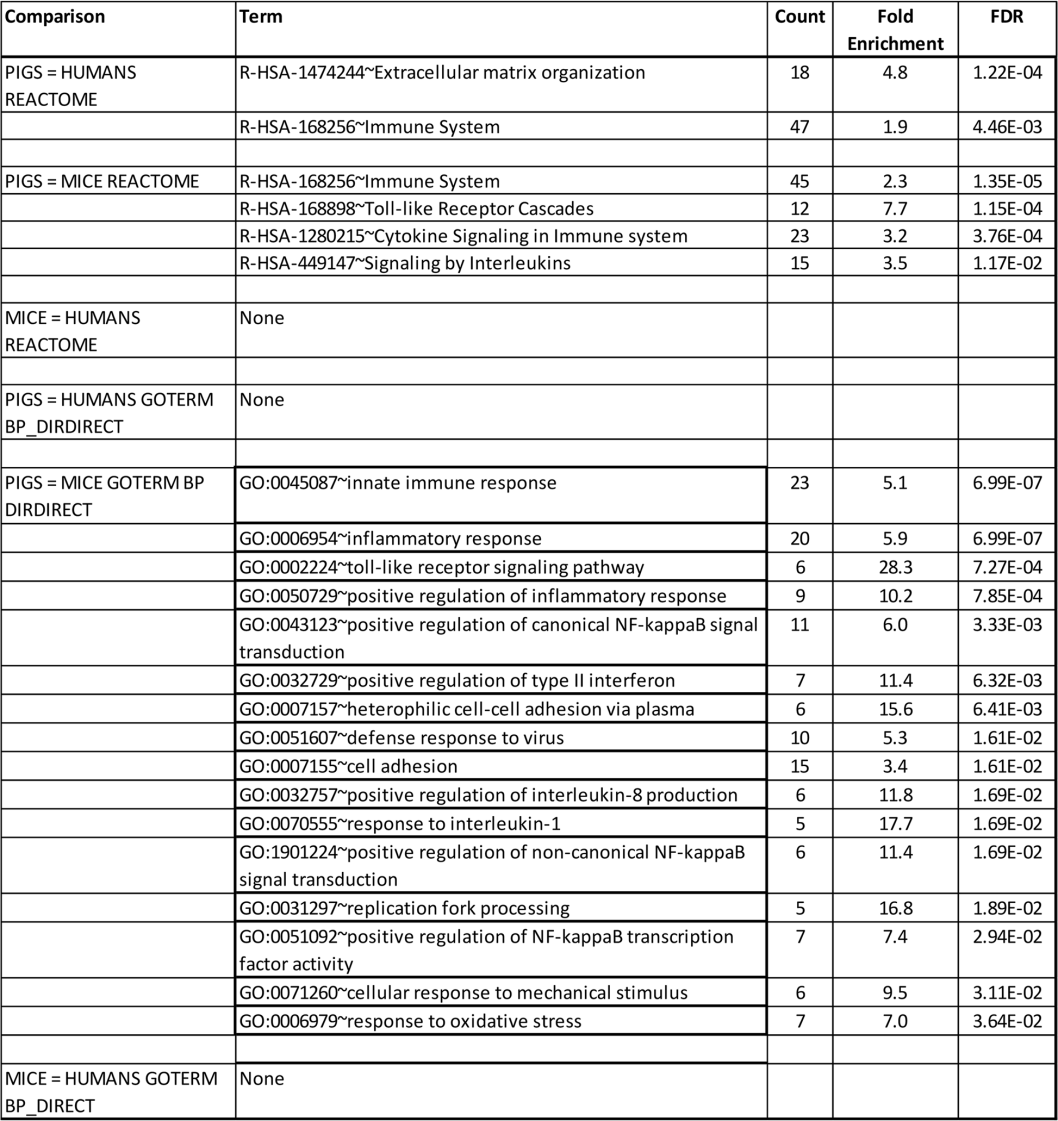
Data Mining of Proteins with Differentially Expressed Functional Domains

We found 3 conserved human-pig KEGG pathways (Tables 5 and 10 S) that were enriched after correction for multiple comparison; hsa04613:Neutrophil extracellular trap formation (4.4 fold, *p* = 3.15E-03), hsa04061:Viral protein interaction with cytokine and cytokine receptor (5.5 fold, *p* = 2.32E-02), hsa05322:Systemic lupus erythematosus (4.3 fold, *p* = 3.42E-02). We also found 3 conserved human-mouse KEGG pathways (Tables 5 and 10 S) that were enriched after correction for multiple comparison; hsa00982:Drug metabolism - cytochrome P450 (12.7 fold, *p* = 3.81E-02), hsa00980:Metabolism of xenobiotics by cytochrome P450 (11.7 fold, *p* = 3.81E-02), and hsa00983:Drug metabolism - other enzymes (11.5 fold, *p* = 3.81E-02).

Similar results were obtained from mining GO BP DIRECT. No enrichment was found for the pig-human comparison; whereas, 17 terms were significant in the pig-mouse comparison; GO:0045087 ~ innate immune response (5.1 fold, *p* = 6.99E-07), GO:0006954 ~ inflammatory response (5.9 fold, *p* = 6.99E-07), GO:0002224 ~ toll-like receptor signaling pathway (28.3 fold, *p* = 7.27E-04), GO:0050729 ~ positive regulation of inflammatory response (10.2 fold, *p* = 7.85E-04), GO:0043123 ~ positive regulation of canonical NF-kappaB signal transduction (6.0 fold, *p* = 3.33E-03), GO:0032729 ~ positive regulation of type II interferon production (11.4 fold, *p* = 6.32E-03), GO:0007157 ~ heterophilic cell-cell adhesion via plasma membrane cell adhesion molecules (15.6 fold, *p* = 6.41E-03), GO:0051607 ~ defense response to virus (5.3 fold, *p* = 1.61E-02), GO:0007155 ~ cell adhesion (3.4 fold, *p* = 1.61E-02), GO:0032757 ~ positive regulation of interleukin-8 production (11.8 fold, *p* = 1.69E-02 ), GO:0070555 ~ response to interleukin-1 (17.7 fold, *p* = 1.69E-02), GO:1,901,224 ~ positive regulation of non-canonical NF-kappaB signal transduction (11.4 fold, *p* = 1.69E-02), GO:0031297 ~ replication fork processing ( 16.8 fold, *p* = 1.89E-02), GO:0051092 ~ positive regulation of NF-kappaB transcription factor activity (7.4 fold, *p* = 2.94E-02), GO:0071260 ~ cellular response to mechanical stimulus (9.5 fold, *p* = 3.11E-02), GO:0006979 ~ response to oxidative stress (7.0 fold, *p* = 3.64E-02). With 2 exceptions, all of these overlapping pathways reflect genes involved in immunity and/or inflammation. No enrichment was observed for the mouse-human comparison.

We evaluated whether the differences in functional domains occurred randomly or whether particular functional domains were more abundant in each species. Of the domains that appear more than 5 times in one species we found that pigs had 6 (Atrophin-1, PHA03307, PHA03378, PRK03918, PRK07764, PTZ00121, PTZ0049) expanded functional domains, mice had 2 (Collagen, Herpes BLFF1, PRK03918) and humans had 5 (Atrophin-1, PHA03307, PHA03378 PTZ00121, PTZ0049) Superfamily domains that were increased or decreased by 25% from the other species. The meaning of this is not clear as the vast majority of these structural domains do not have a defined function.

### C. 5 and 3’UTR and splice variant analyses

#### 1. 5’ and 3’ UTR mRNA conservation

Our current analysis of 8151 mRNAs indicates that 1.2% (98) and 5.09% (415) genes have low conservation of the 5’ or 3’UTR regions, respectively, while 0.85% (69) genes have a combined low 5’ and 3’ conservation (Table 12 S). DAVID REACTOME analysis of these genes indicated that the genes with low 3’ conservation between human and pig, were enriched in genes related to metabolism (19.7 fold) and the Immune System (16.3 fold), although the relationship did not persist after correction for multiple comparisons. Our previous analysis indicates that conservation of mRNA sequences between pigs and humans averages 75%. A separate analysis indicated that the current version of Ensembl build 11.1 does not adequately capture the UTR regions of genes [8].

Next, we determined the potential conservation of 14,122 human transcript variants (NM_+ XM_) in pigs (Table 13 S). We found that pigs could make 89.7% (12,556) of 14,122 human transcript variants. We then examined the NCBI-annotated number of exons for 4,824 randomly selected pig and human genes. We discovered a serious under annotation of exons in the pig genome, almost an exon less per gene with a high bias towards genes with a large number of exons.

## Discussion

The data presented here should help to obtain a finished version of the swine genome and enable improved biological insights. With our manually assembled and annotated non-redundant, 16,146 RNA and 15,613 pig protein sequence library, we assessed the assembly and annotation status of the 3 latest builds of the swine genome and compared them to the mouse and human genomes. Since we generated an extremely large amount of data, we will highlight the major and unique aspects of our analyses.

Our updated annotations, data mining of differentially encoded proteins and pathway enrichments will provide swine researchers with more accurate genomic tools including identifying several hundred pig putative protein-coding genes. Our data confirmed the importance of the swine as a major biomedical model for humans. Our detailed analyses affirmed genomic homologies, with pigs being 5.0 X more likely to have the human gene than mice. Our in-depth work noted that pathway comparisons revealed more pig genes involved in immunity and/or inflammation, thus aiding disease and vaccine efforts.

### Error analysis and categorization

Our comparison of a subset of 6135 protein-coding genes revealed that the percentage of correctly assembled and annotated genes in the NCBI build 11.1, Ensembl build 11.1 and MARC build 1.0 to be 58.9%, 51.7% and 47.1% respectively. The sources of errors were varied but could be systematized to a certain degree: an indel every 12 K, intronless genes, failure to annotate genes for various reasons (intronless genes, mucins, protocadherins, readthrough genes), annotation of endogenous retroviral and retrotransposon sequences as protein-coding genes. In addition, there are genes that are actually missing from one or more genomes. It is likely that the genome build 11.1 is over 95% complete with regard to sequence representation; however, MARC 1.0 may be slightly less complete. One gene, phospholipase A and acyltransferase 4 (PLAAT4), could not be assigned a chromosomal location because it is missing from all 3 genomes (NCBI build 11.1, Ensembl build 11.1 and MARC 1.0). We have determined that it was also missing from several other porcine genomes (Berkshire_pig_v1, Hampshire_pig_v1, Large_White_v1, Pietrain_pig_v1, Tibetan_Pig_v2), but present in the Bamei_pig_v1, Jinhua_pig_v1, Meishan_pig_v1, Rongchang_pig_v1, and Wuzhishan minipig_v1.0 genomes. There are at least two reasons for this. The first is that these DNA regions are difficult to sequence. The second, and more interesting possibility, is that the gene is actually present or absent in different pig breeds. We have determined that the gene is also missing from cows and rodents but is present in other species in the order Artiodactyla, such as *Phacochoerus africanus*, *Diceros bicornis* and *Ceratotherium simum simum*. The protein derived from this gene is a retinoic acid-induced negative regulator of cell proliferation with phospholipase A(1/2) activity [32, 33].

### Errors due to indels

The presence of an indel every 12 Kbp is a major flaw in the Ensembl and NCBI builds of 11.1, affecting the correct assembly and annotation of thousands of genes. and limits the accuracy of splice variant determination as NCBI does not annotate predicted splice variants of low-quality proteins. Although the insertion of an indel by NCBI benefits the analysis of genes affected by a real indel, NCBI also inserts an ambiguous nucleotide (s) and annotates pseudogenes as low-quality proteins in the presence of a predicted stop codon. We previously documented this error for type 1 IFNs [22], CD Markers (BTLA, CD160) [34] and components of the inflammasome (NLRC4, NAIP, NLRP14) [35].

### Keratin associated proteins (KRTAP) and late cornified envelope (LCE) proteins

Keratin associated protein genes are a family of intronless genes that encode type I keratins of the Keratin B2 Superfamily. Our study determined that humans have 124 (107 genes, 17 pseudogenes), pigs have 125 (110 genes and 15 pseudogenes) and mice have 187 (141 genes and 46 pseudogenes) KRTAP genes. A recent census of human genes discovered 93 protein-coding KRTAP genes divided into 26 subfamilies [19]. An expanded KRTAP gene repertoire was previously reported in rodents compared to humans [36]. A preliminary characterization identified 121 KRTAP genes (102 genes and 19 pseudogenes) in pigs [37]; however, the full repertoire of the genes in pigs has not been reported for several reasons. The version of the pig genome used for that characterization (10.2), was missing the 5’ and 3’ region flanking the KRTAP5 cluster [37]; The vast majority of the porcine genes we identified are not annotated genes in NCBI 11.1, Ensembl 11.1 or MARC genomes. Furthermore, they have very limited sequence homology, so assigning 1:1 orthology is difficult.

KRTAP proteins are involved in the formation of intermediate filaments that provide structural support to cells in the body, particularly in the skin and hair [19]. These proteins play a critical role in maintaining the integrity of the skin and hair, and defects in KRTAP genes have been associated with various skin and hair disorders in humans. The number of KRTAP roughly corresponds to the fur coverage of each species [19].

### Vomeronasal receptors (VMRs)

The number of VMRs is proportional to each species reliance on pheromone communication. We found a larger number (225) of mouse VMRs than previous reported [38]; however, some of these may be pseudogenes or may not be expressed.

### Selenoproteins

The discrepancies between the fidelity of selenoprotein assembly and analysis between the species is unknown; but conceptually, it seems possible to automatically assemble and annotate porcine selenoproteins. Selenium, in the form of selenocysteine (Sec) is cotranslationally inserted into polypeptide chains in response to the UGA codon, whose normal function is to terminate translation [39]. To decode UGA as Sec, organisms evolved the Sec insertion machinery that allows incorporation of this amino acid at specific UGA codons in a process requiring a cis-acting, Sec insertion sequence (SECIS) element [39].

### Mucins

The repetitive nature of sequences in mucin genes makes them difficult to assemble [40]. Of the 87 human genes that have their NCBI Annotation category listed as “suggests misassembly”, 8 (MUC1, MUC2, MUC3A, MUC4, MUC5AC, MUC6, MUC8, MUC16, MUC19) are mucins. The actual number of mucin genes in pigs has been difficult to determine. MUCL3 was previously reported to be a pseudogene in pigs [41] but instead is a misassembled gene and a truncated protein in all three builds. Conversely, MUC17 is a pseudogene in pigs. A previous publication reported that this gene is expressed in pigs [5]. Some mucins provide the critical barrier between epithelial cells and the environment. Disorders of mucin synthesis can lead to human diseases like bronchial asthma, ulcerative colitis/inflammatory bowel disease and cystic fibrosis. Having full length pig sequences will aid in the characterization of these mucins in pig models of human disease.

### Protocadherins

The high error rate found for protocadherin assembly and annotation is because the annotation process is not readily amenable to automation. Protocadherins are arranged in tandemly linked gene clusters in a manner similar to that of B-cell and T-cell receptor gene clusters. Virtually nothing is known about pig protocadherins, a single study compared pig, mouse, and human PCDH11X genes [42] and found that all exons present in mouse and pig transcripts had homologous sequences in the human genome but not all exons are represented in human transcripts [42]. Protocadherins play a general role in cell adhesion and development. They are particularly important in the function of neurons. Disruption of protocadherin expression or function is thought to play a role in the development of certain human cancers, neurological and inflammatory diseases [43].

### Readthrough genes

To the best of our knowledge only one pig readthough gene (pig-specific), TNNI2-ACTA1, has been described in the literature [44]. Readthrough transcripts are RNA transcripts that are formed via exon splicing of more than one distinct gene. They are somewhat analogous to alternative splicing in terms of providing genome diversity although their numbers are smaller. An early estimate indicated that there were 751 conjoined or read-through genes in the human genome that are supported by at least one mRNA or EST sequence [45]. More recent GENCODE estimates list 650 and 230 for the human and mouse genome [46]; but there is little overlap between the 2 species. Readthrough protein sequences can be different from their corresponding parent protein sequences due to frame shifting. In addition, a significant number of these are classified as ncRNA, an even smaller subset of these have been designated as nonsense mediated decay (NMD) candidates. Because of the recency of their discovery, the function of readthrough RNAs or proteins is unknown. Genes that are fusions of genes with critical functions in immunity (IFNAR2-IL10RB, TNFSF12-TNFSF13) or metabolism (RBP1-NMNAT3, NT5C1B-RDH14) are likely to have vastly different functions than either parent gene.

### Notable gene superfamilies missing in pigs

The Semenogelin and Seminal Vesicle Secretory Proteins (SVG) Family consists of 2 family members in humans and 6 in mice. Although several catalog vendors claim to have antibodies that are cross reactive to porcine G1 (SEMG1) [18] and an early report describes the presence of a peptide (HNKQEGRDHD) corresponding to human SEMG1 [47] in boar semen, we could not find any SVG family member genes in pigs. The protein is also missing from other determined proteomes of boar semen [48, 49]. The loss of this protein family is not unique to pigs because we could not find any evidence of this protein family members in mammalian orders outside of rodents and primates. The functional meaning of the absence of this protein family is unknown, however, SEMG1 the most abundant protein in human sperm, is essential for sperm coagulation and is broken down to form SgI-29, an antimicrobial peptide [50].

### Splice variant analysis

Our analysis revealed that pigs have the potential to make 89.9% of human transcript variants. To our knowledge there are no recent estimates of this conservation in pig or mice. Early estimates of conservation of splice variants between humans and mice vary widely, from 50% to 70% [51, 52]. An early estimate of conservation between human and pig [53], using ESTs, estimated that around 70% of splice variants were conserved between humans and pigs. We found several instances where automatic annotation led to exon omission. For example, the pig NCBI locus for myelin basic protein (MBP) has one transcript, corresponding to human and mouse isoform 1, but does not include the 3 exons required to make pig splice variants of human and mouse oligodendrocyte lineage (Golli)-Mbp isoforms 1 and 2 [54]. These proteins are represented in the pig TSA archive (Golli-MBP isoform 1 (HDB76269.1, HDA86907.1)) and are partially represented in Ensembl build 11.1 (Golli-MBP isoform 1, ENSSSCP00000055770) and MARC build 1.0 (Golli-MBP isoform 1, ENSSSCP00070023358). The function and expression of Golli-MBPs are distinct from that of MBP and are important for myelin repair [55].

Alternate splicing of genes is a significant source of diversity in the genome. Predicting the number of alternative splice variants has proven to be somewhat difficult. In a previous analysis, Ensembl failed to predict 14 and 20% of validated splice variants in human and mouse genomes, respectively [56]. In the current versions of the pig genomes, the number of pig splice variants is undercounted for several reasons. As previously mentioned, NCBI does not predict splice variants for low-quality proteins (2,807 proteins are annotated as low quality in the NCBI build of 11.1). This set contains a large number of high molecular weight proteins and their contributions to the undercount will be disproportionate because of the large number of nucleotides, and potential splice variants they possess. Irrespective of these errors, the algorithms used to predict splice variants and number of exons, seem to yield vastly different results among the platforms (NCBI versus Ensembl) and species (pigs versus humans or mice). For example, currently the human MBD1 gene in NCBI has 20 exons that can be rearranged to form 165 transcript variants. In Ensembl the human gene has 28 transcript variants. The pig MBD1 gene has 26 exons and 50 predicted splice variants in the NCBI build 11.1, 4 predicted splice variants in Ensembl build 11.1 and 6 in MARC build 1.0. Both the Ensembl build 11.1 and MARC 1.0 loci fail to predict the longest protein-coding transcript. The human PTK2 gene has at least 162 transcript variants; predicted pig splice variants in the NCBI build 11.1, Ensembl build 11.1 and MARC build 1.0 are 40, 8 and 10, respectively. There are numerous other examples of this, but they are beyond the scope of the current manuscript.

Splice variants can give rise to identical proteins or can lead to distinct isoforms. Although it has been suggested that most proteins have a single isoform [57] and that many splice variants are not translated into proteins, there are multiple splice variants for 72% of annotated human genes and 205,000 transcripts had protein-coding potential (> 10 transcripts per gene). Predicted or actual functional consequences of each splice variant are incomplete, even for human and mouse genes. A full discussion of this is beyond the scope of the current manuscript. Instead, we will provide a few examples of differential splice variants or isoforms where there is comparative, functional data.

Pigs and humans can make all 17 transcript variants/isoforms of the T cell transcription factor, TCF7L2, involved with antiviral responses [18]. In contrast pigs can only make 1 out of 5 isoforms of the three prime repair exonuclease 1 (TREX1) and 1 out of 4 isoforms of interferon regulatory factor 9 (IRF9), proteins involved in antiviral immune responses [19]. Humans express 3 isoforms of interleukin 22 receptor, alpha 2 (IL22RA2) that differ in expression and/or function [58]. Pigs and mice lack exon 3, that gives rise to IL22RA2 isoform 1 in humans, and can only form the soluble form (isoform 2) [59]. Alternate splice forms of LY96 (MD-2) that inhibit signaling have been identified in both mice and humans, but these alternate splice forms arise from different splicing events. The mouse protein isoform MD-2B, formed by a 54 base pair deletion at the 5’ end of exon 3, inhibits TLR4 activation by LPS [60]. The human isoform 2 (MD-2s), formed by skipping exon 2, also inhibits LPS signaling through TLR4 and is not found in mice [61]. We predict isoform 2 can be formed in pigs but found no evidence for isoform 2 expression in the EST and TSA archives. NCBI predicted that isoform 2 occurs in various Canids (*Canis lupus familiaris*, *Canis lupus dingo*, *Vulpes lagopus*) and Pinnipeds (*Neomonachus schauinslandi*, *Mirounga angustirostris*, *Mirounga leonine*, *Halichoerus grypu*).

## Conclusions

In this manuscript, we analyzed Ensembl and NCBI builds 11.1 and MARC 1.0, with a large sequence library of manually assembled and annotated RNA and protein sequences, in order to improve the annotation of the pig genome and discover systematic sources of errors. These sources include a frequently occurring indel in proteins of large size that alter the predicted size or delegation of a gene as protein-coding. This leads to failure to properly assemble large sized genes, e.g., the mucin and protocadherin genes. Additional errors, almost exclusive to Ensembl build 11.1 and MARC build 1.0 include selenoprotein genes being assigned a premature stop codon and numerous retrotransposon (RT and TR) and retroviral genes being assigned protein coding status. We also identified several hundred pig putative protein-coding genes that are missing from one or more genome builds in the process.

We analyzed the conservation of pig and human 5’ and 3’ UTR RNA regions and RNA splice variants. We assembled a partial, but nonredundant and highly annotated, pig RNAome and proteome and used it to identify 1–1 mouse and/or human orthologs. We compared the 1–1 orthologs or proteins with shared or non-shared functional domains, for all 3 species, to determine functional enrichment. The results are summarized in Table 7. These data overwhelmingly support the relevance and importance of the pig as a biomedical research model for humans. Our analysis also highlights areas where mice may be a better model and areas where both of these species are likely to be of limited use. For example, although we have made a strong case for the use of the pig as a biomedical model, particularly in the area of nutrition and immunity, our DAVID REACTOME analysis of genes with low 3’ conservation between human and pig showed enrichment in genes related to metabolism and the immune system. The 3’ UTR of mRNA can contain binding sites for regulatory RNA and proteins.

**Table 7:**
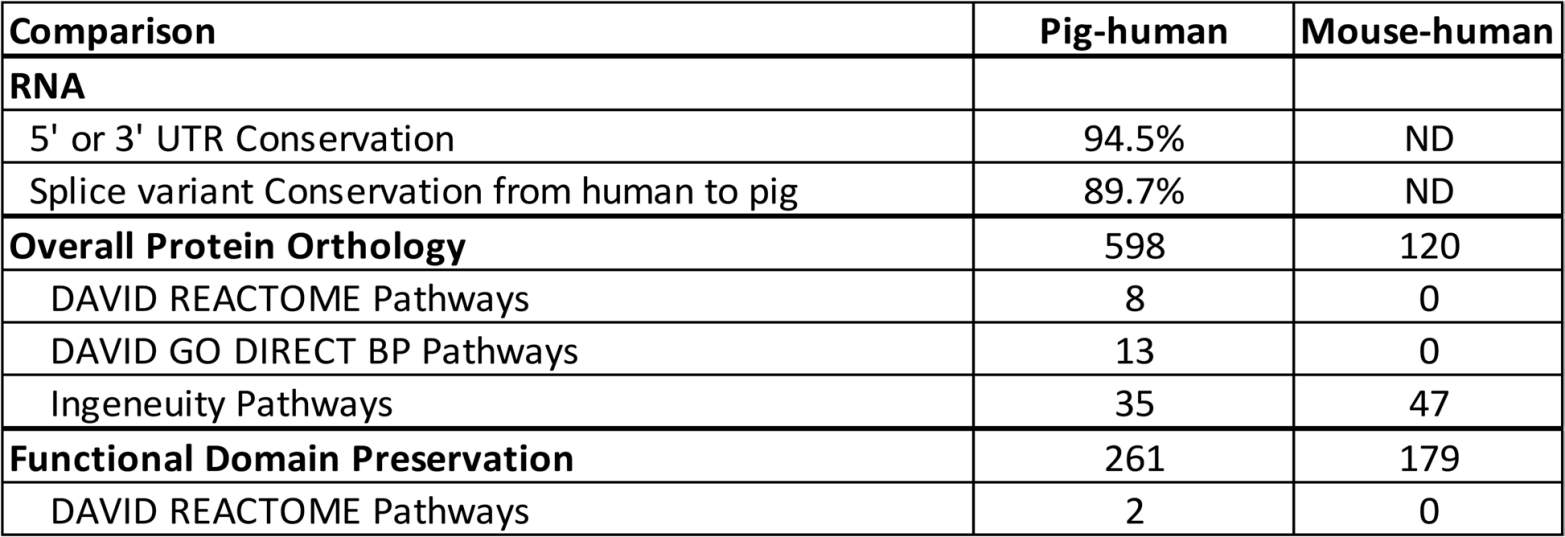
Summation of Results

In addition to these comparisons, we provide the first description and evidence for over 100 potential porcine read through genes, the first formal identification (to our knowledge) of pig Golli-MBPs and a complete, comparative analysis of pig, mouse and human VMR receptors.

One of the strengths of our approach is that we identify pig transcript variants and protein isoforms based upon their orthology to human counterparts. This would make the genome annotation process better align with the human genome and make potential functions of known human transcripts translatable to the pig. The need to better align the nomenclature of species used in biomedical research to their human counterpart has recently been emphasized [44]. One of the weaknesses of our approach is that we did not attempt to identify pig-specific transcripts. Furthermore, we did not characterize mouse transcripts. Another weakness is that for protein functional domain analysis, we used results from the NCBI BLAST search. While this program identifies macrodomains, it cannot map certain fine structures of proteins as we have done in our previous analysis [35].

There are currently more than 31 sequenced pig genomes that are publicly available. The annotation states of these are highly variable. Development of more accurate, artificial intelligence-based annotation software is urgently needed. We propose using our templates and nomenclature system to train such software for use in any current or future generation of pig genomes.

## Materials and methods

One-to-one orthology was determined for pig-mouse, pig-human or mouse-human genes as previously described [16]. Briefly reciprocal cross-BLASTing of pig (*Sus scrofa*) sequence sources in GenBank (non-redundant, expressed sequences tag, high throughput genomic sequence, whole genome shotgun contig sequences (WGS), transcriptome shot gun assembly (TSA) and expressed sequence tag (EST)) was performed using discontiguous Megablast (default settings, word size = 11), using reference sequence accession numbers to human or mouse genes/proteins of interest. Ensembl build 11.1 (release 111 - Jan 2024) and Ensembl MARC build 1.0 (release 111 - Jan 2024) were searched using the default settings. When 1–1 orthology could not be established for pig genes based upon protein homology, the RNA was used. If orthology could not be determined by protein and RNA homology, the relative chromosomal location was used to determine orthology [22]. The human gene symbol was assigned for pig orthologs whenever appropriate. For pig-specific paralogs, the gene symbol assigned was based upon comparative homology to the human gene, following the convention of the human gene family. The gene symbol was then terminated with an asterisk (*). For example, pig-specific paralogs of human SLC7A3, were assigned SLC7A3L1*, SLC7A3L2*, SLC7A3L3*, etc. with SLC7A3L1 being the closest in homology. Splice variant/exon conservation of the pig gene was determined relative to the human reference transcript. Pig-specific transcript variants were not determined.

Specifically, our classifications in Table 2 followed these BLASTing protocols: (1) Error in annotated locus: BLASTing Full Length Sequence into Ensembl or NCBI Gene Databases or a previous determination of an indel made by the NCBI annotation pipeline. Alternatively, the predicted protein sequences not of correct length to due mis assembly or the annotated NCBI reference RNA/protein sequences are not full length in Ensembl. (2) Split into multiple loci: BLASTing Full Length Sequence into Ensembl or NCBI Gene Databases; Gene appears in 2 or more loci and is not antisense RNA. (3) Multiple genes one locus: BLASTing Full Length Sequence into Ensembl or NCBI Gene Databases; Locus contains 2 or more distinct protein sequences. Sequences may be antisense RNA or protein transcribed from the opposite allele. (4) Not an annotated gene : BLASTing Full Length Sequence into Ensembl or NCBI Gene Databases Sequence is present in the genome but is not annotated as a gene locus. (5) Sequence is not present: BLASTing Full Length Sequence into Ensembl or NCBI Gene Databases Sequence is not present in the genome but may be annotated as a gene locus if the gene had been sequences previous tor in addition to the genome assembly.

To determine retrovirus or retrotransposase gene contamination of the protein coding genome, PERVA (HQ540592.1), PERVB (AY099324.1), PERVC (OR574834.1), reverse transcriptase (ABR01162.1) and transposase (ABR01161.1) sequences were mapped to builds 11.1 of the Ensembl 11.1 and NCBI build 11.1 as well as versions 1 of the Ensembl-curated Bamei, Berkshire, Hampshire, Jinhua, Meishan, Landrace, Large White, Ossabaw Pietrain, Rongchang, Tibetan, Wuzhishan genomes. Data for Ensembl 11.1 and NCBI build 11.1 are shown in Table 4S.

Predicted mRNAs were analyzed for errors (ambiguous nucleotides, gene duplications artifacts, mis-assemblies, mis-annotations). Whenever an ambiguous nucleotide was assigned by NCBI, we blasted the sequence against the WGS, TSA and EST as well as versions 1 of the Ensembl-curated Bamei, Berkshire, Hampshire, Jinhua, Meishan, Landrace, Large White, Ossabaw Pietrain, Rongchang, Tibetan, Wuzhishan genomes databases, to obtain a consensus sequence. Predicted mRNAs were translated into proteins using the ExPASy translate tool (http://web.expasy.org/translate/). The size (in amino acids) of the major protein isoforms was used as a checksum to determine whether the respective genome assembly was correct.

Potential porcine readthrough genes were identified by comparison to human and mouse readthrough genes. A predicative scoring system was developed based on whether the transcript exists in other species and whether there was evidence that pigs can make the respective transcript (sequence found in TSA, EST, NCBI and Ensembl build 11.1 and MARC 1.0) databases. Pig-specific readthroughs were distinguished from chimeric artifacts by determining whether the transcript appears in other species and if there was support for the transcript (previously sequenced RNA). Consensus sequences were numerically annotated with base pair positions aligning to the beginning and end of human reference transcript. where possible. Conservation of the 5’ and 3’ predicted pig mRNA was then determined relative to the human reference transcript. Non-coding RNA will not be discussed here with the exception of small nucleolar RNAs (snoRNAs) and small Cajal body-specific RNAs (ScaRNAs).

We determined 1 to 1 to 1 pig-mouse-human orthology for 12,720, 12,887 and 12,770 protein-coding genes in pigs, mice and humans, respectively (total 38,377). We excluded olfactory receptors (ORs), T and B Cell receptors (TCR and BCR), and MHC class I and II proteins from our analysis and discussion because determining 1:1 orthology for these genes is difficult. The human and mouse genome nomenclature committees have adopted different conventions for assigning nomenclature and assigning orthology is not straightforward (cannot easily be determined by reciprocal cross blasting and/or chromosomal location). These genes will be described in separate manuscripts. A similar situation exists with regard to TRs. To determine structural orthology of TRs, we conducted phylogenetic tree analysis using Geneious Prime program (Geneious Pro v 20231.2) and the Jukes–Cantor algorithm. These unambiguous protein orthologs (from 3 species) were used for Venn analysis using Venny 2.1. (http://bioinfogp.cnb.csic.es/tools/venny/index.html). One to one pig/human, pig/mouse or mouse/human orthologs were analyzed by DAVID (https://davidbioinformatics.nih.gov/ ), REACTOME (https://reactome.org/) and Go Direct BP (https://geneontology.org/) databases and queried to determine functional enrichment. Alternatively, we used our highly annotated database to determine whether the protein was part of the immunome [13] or involve in nutrition and/or metabolism [16]. Differentially encoded genes were also compared using Ingenuity Pathway Analysis (IPA) software (QIAGEN Bioinformatics, Redwood, CA). Conservation of protein functional domains was determined by comparing the BLAST graphic summary of the longest, comparable protein isoform sequences. Proteins with non or shared domains were analyzed by DAVID to determine functional enrichment.

## Supplementary Information

Supplementary Material 1: Table S1. Summary of Gene Omission Errors.

Supplementary Material 2: Table S2. Analysis of Proteins of Extreme Size.

Supplementary Material 3: Table S3. Status of KRTAP and LCE Genes in the 3 Pig Genomes.

Supplementary Material 4: Table S4. Status of Retrotransposons and Retroviruses in the 3 Pig Genomes.

Supplementary Material 5: Table S5. Summary of Build Errors in Selenoprotein Genes.

Supplementary Material 6: Table S6. Status of Mucin Genes in the 3 Pig Genomes .

Supplementary Material 7: Table S7. Status of Protocadherin Genes in the 3 Pig Genomes.

Supplementary Material 8: Table S8. Status of Readthrough Genes in the 3 Pig Genomes.

Supplementary Material 9: Table S9. Microproteins.

Supplementary Material 10: Table S10. Pig, Mouse and Human Gene and Total Protein Comparisons.

Supplementary Material 11: Table S11. Analyses of Structural Domain Comparisons.

Supplementary Material 12: Table S12. Comparison of Genes with Low 5' and or 3 Conservation Between Humans and Pigs.

Supplementary Material 13: Table S13. Summary of Conserved Swine/Human Splice Variants.

## Data Availability

All data generated or analyzed during this study are included in this published article [and its supplementary information files]. Complete data (pig RNA and protein sequences and their respective annotations) for these analyses can be found in our online database (http://tinyurl.com/hxxq3ur).

## Abbreviations

AA: Amino Acid
BCR: B Cell receptor
ERV: Endogenous retrovirus
EST: Expressed sequence tag
HAVANA: Human and Vertebrate Analysis and Annotation
KEGG: Kyoto Encyclopedia of Genes and Genomes
KRTAP: Keratin associated proteins
LCE: Late cornified envelope
miRNA: MicroRNA
N: Nucleotides
ncRNA: Non-coding RNA
NMD: Nonsense mediated decay
OR: Olfactory Receptor
ScaRNA: Small Cajal body-specific RNA)
SEP: SORF-encoded protein
SnoRNAs: Small nucleolar RNA
sORFs: Small open reading frame
RT: Reverse transcriptase
Sec: Selenocysteine
SRA: Short Read Archive Database
tblastn: Translated BLAST
TSA: Transcriptome Shotgun Assembly Sequence Database
TR: Transposase
UTR: Untranslated region
VMR: Vomeronasal Receptors
WGS: Whole genome shotgun contig sequences

## References

[1] Mudge JM, Harrow J. Creating reference gene annotation for the mouse C57BL6/J genome assembly. Mamm Genome. 2015;26(9–10):366–78. 26187010 10.1007/s00335-015-9583-x PMC4602055

[2] Nurk S, Koren S, Rhie A, Rautiainen M, Bzikadze AV, Mikheenko A, Vollger MR, Altemose N, Uralsky L, Gershman A, et al. The complete sequence of a human genome. Science. 2022;376(6588):44–53. 35357919 10.1126/science.abj6987 PMC9186530

[3] Liu J, Li Q, Hu Y, Yu Y, Zheng K, Li D, Qin L, Yu X. The complete telomere-to-telomere sequence of a mouse genome. Science. 2024;386(6726):1141–6. 39636971 10.1126/science.adq8191

[4] George CM, Burrowes V, Perin J, Oldja L, Biswas S, Sack D, Ahmed S, Haque R, Bhuiyan NA, Parvin T, et al. Enteric infections in young children are associated with environmental enteropathy and impaired growth. Trop Med Int Health. 2018;23(1):26–33. 29121442 10.1111/tmi.13002

[5] Gilbert DG. Genes of the pig, Sus scrofa, reconstructed with evidentialgene. PeerJ. 2019;7:e6374. 30723633 10.7717/peerj.6374 PMC6361002

[6] Summers KM, Bush SJ, Wu C, Su AI, Muriuki C, Clark EL, Finlayson HA, Eory L, Waddell LA, Talbot R, et al. Functional annotation of the transcriptome of the Pig, Sus scrofa, based upon network analysis of an RNAseq transcriptional atlas. Front Genet. 2019;10:1355. 32117413 10.3389/fgene.2019.01355 PMC7034361

[7] Beiki H, Liu H, Huang J, Manchanda N, Nonneman D, Smith TPL, Reecy JM, Tuggle CK. Improved annotation of the domestic pig genome through integration of Iso-Seq and RNA-seq data. BMC Genomics. 2019;20(1):344. 31064321 10.1186/s12864-019-5709-y PMC6505119

[8] Warr A, Affara N, Aken B, Beiki H, Bickhart DM, Billis K, Chow W, Eory L, Finlayson HA, Flicek P et al. An improved pig reference genome sequence to enable pig genetics and genomics research. Gigascience. 2020;9(6);GIAA051. 10.1093/gigascience/giaa051. 10.1093/gigascience/giaa051 PMC7448572 32543654

[9] van der Hee B, Madsen O, Vervoort J, Smidt H, Wells JM. Congruence of transcription programs in adult stem Cell-Derived jejunum organoids and original tissue during Long-Term culture. Front Cell Dev Biol. 2020;8:375. 32714922 10.3389/fcell.2020.00375 PMC7343960

[10] Yu W, Moninger TO, Thurman AL, Xie Y, Jain A, Zarei K, Powers LS, Pezzulo AA, Stoltz DA, Welsh MJ. Cellular and molecular architecture of submucosal glands in wild-type and cystic fibrosis pigs. Proc Natl Acad Sci U S A. 2022;119(4):e2119759119. 10.1073/pnas.2119759119. 10.1073/pnas.2119759119 PMC8794846 35046051

[11] Dawson HD. A comparative assessment of the pig, mouse and human genomes. The minipig in biomedical research. Boca Raton, FL: CRC; 2011. pp. 323–42.

[12] Groenen MA, Archibald AL, Uenishi H, Tuggle CK, Takeuchi Y, Rothschild MF, Rogel-Gaillard C, Park C, Milan D, Megens HJ, et al. Analyses of pig genomes provide insight into Porcine demography and evolution. Nature. 2012;491(7424):393–8. 23151582 10.1038/nature11622 PMC3566564

[13] Dawson HD, Loveland JE, Pascal G, Gilbert JG, Uenishi H, Mann KM, Sang Y, Zhang J, Carvalho-Silva D, Hunt T, et al. Structural and functional annotation of the Porcine immunome. BMC Genomics. 2013;14:332. 23676093 10.1186/1471-2164-14-332 PMC3658956

[14] Doncheva NT, Palasca O, Yarani R, Litman T, Anthon C, Groenen MAM, Stadler PF, Pociot F, Jensen LJ, Gorodkin J. Human pathways in animal models: possibilities and limitations. Nucleic Acids Res. 2021;49(4):1859–71. 33524155 10.1093/nar/gkab012 PMC7913694

[15] Triant DA, Walsh AT, Hartley GA, Petry B, Stegemiller MR, Nelson BM, McKendrick MM, Fuller EP, Cockett NE, Koltes JE, et al. AgAnimalGenomes: browsers for viewing and manually annotating farm animal genomes. Mamm Genome. 2023;34(3):418–36. 37460664 10.1007/s00335-023-10008-1 PMC10382368

[16] Dawson HD, Chen C, Gaynor B, Shao J, Urban JF Jr. The Porcine translational research database: a manually curated, genomics and proteomics-based research resource. BMC Genomics. 2017;18(1):643. 28830355 10.1186/s12864-017-4009-7 PMC5568366

[17] Grzybowska EA. Human intronless genes: functional groups, associated diseases, evolution, and mRNA processing in absence of splicing. Biochem Biophys Res Commun. 2012;424(1):1–6. 22732409 10.1016/j.bbrc.2012.06.092

[18] Jorquera R, Gonzalez C, Clausen P, Petersen B, Holmes DS. Improved ontology for eukaryotic single-exon coding sequences in biological databases. Database (Oxford). 2018; 2018:1–6. 10.1093/database/bay089 PMC6146118 30239665

[19] Litman T, Stein WD. Ancient lineages of the keratin-associated protein (KRTAP) genes and their co-option in the evolution of the hair follicle. BMC Ecol Evol. 2023;23(1):7. 36941546 10.1186/s12862-023-02107-z PMC10029157

[20] Podvalnyi A, Kopernik A, Sayganova M, Woroncow M, Zobkova G, Smirnova A, Esibov A, Deviatkin A, Volchkov P, Albert E. Quantitative analysis of Pseudogene-Associated errors during germline variant calling. Int J Mol Sci. 2025;26(1);363. 10.3390/ijms26010363. 10.3390/ijms26010363 PMC11719938 39796219

[21] Sharma M, Arora I, Stoll ML, Li Y, Morrow CD, Barnes S, Berryhill TF, Li SZ, Tollefsbol TO. Nutritional combinatorial impact on the gut microbiota and plasma short-chain fatty acids levels in the prevention of mammary cancer in Her2/neu Estrogen receptor-negative Transgenic mice. PLoS ONE. 2020;15(12):e0234893. 10.1371/journal.pone.0234893. 10.1371/journal.pone.0234893 PMC7774855 33382695

[22] Dawson HD, Sang Y, Lunney JK. Porcine cytokines, chemokines and growth factors: 2019 update. Res Vet Sci. 2020;131:266–300. 32442727 10.1016/j.rvsc.2020.04.022

[23] Zong W, Zhao R, Wang X, Zhou C, Wang J, Chen C, Niu N, Zheng Y, Chen L, Liu X, et al. Population genetic analysis based on the polymorphisms mediated by transposons in the genomes of pig. DNA Res. 2024;31(2):dsae008. 38447059 10.1093/dnares/dsae008 PMC11090087

[24] Hancks DC, Kazazian HH Jr. Active human retrotransposons: variation and disease. Curr Opin Genet Dev. 2012;22(3):191–203. 22406018 10.1016/j.gde.2012.02.006 PMC3376660

[25] Sironen A, Vilkki J, Bendixen C, Thomsen B. Infertile Finnish Yorkshire boars carry a full-length LINE-1 retrotransposon within the KPL2 gene. Mol Genet Genomics. 2007;278(4):385–91. 17610085 10.1007/s00438-007-0256-7

[26] Niebert M, Rogel-Gaillard C, Chardon P, Tonjes RR. Characterization of chromosomally assigned replication-competent gamma Porcine endogenous retroviruses derived from a large white pig and expression in human cells. J Virol. 2002;76(6):2714–20. 11861838 10.1128/JVI.76.6.2714-2720.2002 PMC136001

[27] Ishihara S, Kumagai M, Arakawa A, Taniguchi M, Cuc NTK, Pham LD, Mikawa S, Kikuchi K. Detection of non-reference Porcine endogenous retrovirus loci in the Vietnamese native pig genome. Sci Rep. 2022;12(1):10485. 35729348 10.1038/s41598-022-14654-4 PMC9213404

[28] Simpson J, Kozak CA, Boso G. Evolutionary conservation of an ancient retroviral Gagpol gene in artiodactyla. J Virol. 2023;97(9):e0053523. 37668369 10.1128/jvi.00535-23 PMC10537755

[29] Griffiths DJ. Endogenous retroviruses in the human genome sequence. Genome Biol. 2001;2(6):REVIEWS1017. 11423012 10.1186/gb-2001-2-6-reviews1017 PMC138943

[30] Leong AZ, Lee PY, Mohtar MA, Syafruddin SE, Pung YF, Low TY. Short open reading frames (sORFs) and microproteins: an update on their identification and validation measures. J Biomed Sci. 2022;29(1):19. 35300685 10.1186/s12929-022-00802-5 PMC8928697

[31] Leblanc S, Yala F, Provencher N, Lucier JF, Levesque M, Lapointe X, Jacques JF, Fournier I, Salzet M, Ouangraoua A, et al. OpenProt 2.0 builds a path to the functional characterization of alternative proteins. Nucleic Acids Res. 2024;52(D1):D522–8. 37956315 10.1093/nar/gkad1050 PMC10767855

[32] DiSepio D, Ghosn C, Eckert RL, Deucher A, Robinson N, Duvic M, Chandraratna RA, Nagpal S. Identification and characterization of a retinoid-induced class II tumor suppressor/growth regulatory gene. Proc Natl Acad Sci U S A. 1998;95(25):14811–5. 9843971 10.1073/pnas.95.25.14811 PMC24531

[33] Uyama T, Jin XH, Tsuboi K, Tonai T, Ueda N. Characterization of the human tumor suppressors TIG3 and HRASLS2 as phospholipid-metabolizing enzymes. Biochim Biophys Acta. 2009;1791(12):1114–24. 19615464 10.1016/j.bbalip.2009.07.001

[34] Dawson HD, Lunney JK. Porcine cluster of differentiation (CD) markers 2018 update. Res Vet Sci. 2018;118:199–246. 29518710 10.1016/j.rvsc.2018.02.007

[35] Dawson HD, Smith AD, Chen C, Urban JF Jr. An in-depth comparison of the Porcine, murine and human inflammasomes; lessons from the Porcine genome and transcriptome. Vet Microbiol. 2017;202:2–15. 27321134 10.1016/j.vetmic.2016.05.013

[36] Wu DD, Irwin DM, Zhang YP. Molecular evolution of the keratin associated protein gene family in mammals, role in the evolution of mammalian hair. BMC Evol Biol. 2008;8:241. 18721477 10.1186/1471-2148-8-241 PMC2528016

[37] Khan I, Maldonado E, Vasconcelos V, O’Brien SJ, Johnson WE, Antunes A. Mammalian keratin associated proteins (KRTAPs) subgenomes: disentangling hair diversity and adaptation to terrestrial and aquatic environments. BMC Genomics. 2014;15(1):779. 25208914 10.1186/1471-2164-15-779 PMC4180150

[38] Rodriguez I, Del Punta K, Rothman A, Ishii T, Mombaerts P. Multiple new and isolated families within the mouse superfamily of V1r vomeronasal receptors. Nat Neurosci. 2002;5(2):134–40. 11802169 10.1038/nn795

[39] Labunskyy VM, Hatfield DL, Gladyshev VN. Selenoproteins: molecular pathways and physiological roles. Physiol Rev. 2014;94(3):739–77. 24987004 10.1152/physrev.00039.2013 PMC4101630

[40] Lang T, Pelaseyed T. Discovery of a MUC3B gene reconstructs the membrane mucin gene cluster on human chromosome 7. PLoS ONE. 2022;17(10):e0275671. 36256656 10.1371/journal.pone.0275671 PMC9578598

[41] Shigenari A, Ando A, Renard C, Chardon P, Shiina T, Kulski JK, Yasue H, Inoko H. Nucleotide sequencing analysis of the swine 433-kb genomic segment located between the non-classical and classical SLA class I gene clusters. Immunogenetics. 2004;55(10):695–705. 14673549 10.1007/s00251-003-0627-0

[42] Blanco-Arias P, Sargent CA, Affara NA. A comparative analysis of the pig, mouse, and human PCDHX genes. Mamm Genome. 2004;15(4):296–306. 15112107 10.1007/s00335-003-3034-9

[43] Pancho A, Aerts T, Mitsogiannis MD, Seuntjens E. Protocadherins at the crossroad of signaling pathways. Front Mol Neurosci. 2020;13:117. 32694982 10.3389/fnmol.2020.00117 PMC7339444

[44] Liu D, Xia J, Yang Z, Zhao X, Li J, Hao W, Yang X. Identification of chimeric RNAs in pig skeletal muscle and transcriptomic analysis of chimeric RNA TNNI2-ACTA1 V1. Front Vet Sci. 2021;8:742593. 34778431 10.3389/fvets.2021.742593 PMC8578878

[45] Prakash T, Sharma VK, Adati N, Ozawa R, Kumar N, Nishida Y, Fujikake T, Takeda T, Taylor TD. Expression of conjoined genes: another mechanism for gene regulation in eukaryotes. PLoS ONE. 2010;5(10):e13284. 20967262 10.1371/journal.pone.0013284 PMC2953495

[46] Frankish A, Carbonell-Sala S, Diekhans M, Jungreis I, Loveland JE, Mudge JM, Sisu C, Wright JC, Arnan C, Barnes I, et al. GENCODE: reference annotation for the human and mouse genomes in 2023. Nucleic Acids Res. 2023;51(D1):D942–9. 36420896 10.1093/nar/gkac1071 PMC9825462

[47] Jonakova V, Kraus M, Veselsky L, Cechova D, Bezouska K, Ticha M. Spermadhesins of the AQN and AWN families, DQH sperm surface protein and HNK protein in the heparin-binding fraction of Boar seminal plasma. J Reprod Fertil. 1998;114(1):25–34. 9875152 10.1530/jrf.0.1140025

[48] Perez-Patino C, Parrilla I, Li J, Barranco I, Martinez EA, Rodriguez-Martinez H, Roca J. The proteome of pig spermatozoa is remodeled during ejaculation. Mol Cell Proteom. 2019;18(1):41–50. 10.1074/mcp.RA118.000840 PMC6317480 30257877

[49] Xu Y, Han Q, Ma C, Wang Y, Zhang P, Li C, Cheng X, Xu H. Comparative proteomics and phosphoproteomics analysis reveal the possible breed difference in Yorkshire and duroc Boar spermatozoa. Front Cell Dev Biol. 2021;9:652809. 34336820 10.3389/fcell.2021.652809 PMC8322956

[50] Zhao A, Urban JF Jr., Anthony RM, Sun R, Stiltz J, van Rooijen N, Wynn TA, Gause WC, Shea-Donohue T. Th2 cytokine-induced alterations in intestinal smooth muscle function depend on alternatively activated macrophages. Gastroenterology. 2008;135(1):217–e225211. 18471439 10.1053/j.gastro.2008.03.077 PMC2954589

[51] Modrek B, Lee CJ. Alternative splicing in the human, mouse and rat genomes is associated with an increased frequency of exon creation and/or loss. Nat Genet. 2003;34(2):177–80. 12730695 10.1038/ng1159

[52] Nurtdinov RN, Artamonova II, Mironov AA, Gelfand MS. Low conservation of alternative splicing patterns in the human and mouse genomes. Hum Mol Genet. 2003;12(11):1313–20. 12761046 10.1093/hmg/ddg137

[53] Nygard AB, Cirera S, Gilchrist MJ, Gorodkin J, Jorgensen CB, Fredholm M. A study of alternative splicing in the pig. BMC Res Notes. 2010;3:123. 20444244 10.1186/1756-0500-3-123 PMC2882375

[54] Campagnoni AT, Pribyl TM, Campagnoni CW, Kampf K, Amur-Umarjee S, Landry CF, Handley VW, Newman SL, Garbay B, Kitamura K. Structure and developmental regulation of Golli-mbp, a 105-kilobase gene that encompasses the Myelin basic protein gene and is expressed in cells in the oligodendrocyte lineage in the brain. J Biol Chem. 1993;268(7):4930–8. 7680345

[55] Siu CR, Balsor JL, Jones DG, Murphy KM. Classic and Golli Myelin basic protein have distinct developmental trajectories in human visual cortex. Front Neurosci. 2015;9:138. 25964736 10.3389/fnins.2015.00138 PMC4408849

[56] Tapial J, Ha KCH, Sterne-Weiler T, Gohr A, Braunschweig U, Hermoso-Pulido A, Quesnel-Vallieres M, Permanyer J, Sodaei R, Marquez Y, et al. An atlas of alternative splicing profiles and functional associations reveals new regulatory programs and genes that simultaneously express multiple major isoforms. Genome Res. 2017;27(10):1759–68. 28855263 10.1101/gr.220962.117 PMC5630039

[57] Gonzalez-Porta M, Frankish A, Rung J, Harrow J, Brazma A. Transcriptome analysis of human tissues and cell lines reveals one dominant transcript per gene. Genome Biol. 2013;14(7):R70. 23815980 10.1186/gb-2013-14-7-r70 PMC4053754

[58] Gomez-Fernandez P, Urtasun A, Paton AW, Paton JC, Borrego F, Dersh D, Argon Y, Alloza I, Vandenbroeck K. Long Interleukin-22 binding protein isoform-1 is an intracellular activator of the unfolded protein response. Front Immunol. 2018;9:2934. 30619294 10.3389/fimmu.2018.02934 PMC6302113

[59] Weiss B, Wolk K, Grunberg BH, Volk HD, Sterry W, Asadullah K, Sabat R. Cloning of murine IL-22 receptor alpha 2 and comparison with its human counterpart. Genes Immun. 2004;5(5):330–6. 15201862 10.1038/sj.gene.6364104

[60] Ohta S, Bahrun U, Tanaka M, Kimoto M. Identification of a novel isoform of MD-2 that downregulates lipopolysaccharide signaling. Biochem Biophys Res Commun. 2004;323(3):1103–8. 15381113 10.1016/j.bbrc.2004.08.203

[61] Gray P, Michelsen KS, Sirois CM, Lowe E, Shimada K, Crother TR, Chen S, Brikos C, Bulut Y, Latz E, et al. Identification of a novel human MD-2 splice variant that negatively regulates Lipopolysaccharide-induced TLR4 signaling. J Immunol. 2010;184(11):6359–66. 20435923 10.4049/jimmunol.0903543 PMC3057206

